# Amyloid-β_1–43_ cerebrospinal fluid levels and the interpretation of *APP*, *PSEN1* and *PSEN2* mutations

**DOI:** 10.1186/s13195-020-00676-5

**Published:** 2020-09-11

**Authors:** Federica Perrone, Maria Bjerke, Elisabeth Hens, Anne Sieben, Maarten Timmers, Arne De Roeck, Rik Vandenberghe, Kristel Sleegers, Jean-Jacques Martin, Peter P. De Deyn, Sebastiaan Engelborghs, Julie van der Zee, Christine Van Broeckhoven, Rita Cacace, on behalf of the BELNEU Consortium, Johan Goeman, Roeland Crols, Bart Dermaut, Adrian Ivanoiu, Bernard Hanseeuw, Olivier Deryck, Bruno Bergmans, Jan Versijpt, Janssen Pharmaceutica, Takaomi Saido

**Affiliations:** 1 Neurodegenerative Brain Diseases Group, VIB Center for Molecular Neurology, Antwerp, Belgium; 2 Institute Born-Bunge, Antwerp, Belgium; 3 Department of Biomedical Sciences, University of Antwerp, Antwerp, Belgium; 4 Reference Centre for Biological Markers of Dementia (BIODEM), Institute Born-Bunge, University of Antwerp, Antwerp, Belgium; 5 Laboratory of Neurochemistry and Center for Neurosciences, UZ Brussel and Vrije Universiteit Brussel, Brussels, Belgium; 6 Department of Neurology and Memory Clinic, Hospital Network Antwerp, Middelheim and Hoge Beuken, Antwerp, Belgium; 7 Department of Neurology, University Hospital Antwerp, Edegem, Belgium; 8 Department of Neurology, University Hospital Brussel and Center for Neurosciences, Vrije Universiteit Brussel, Brussels, Belgium; 9 Department of Neurology, University Hospital Ghent and University of Ghent, Ghent, Belgium; 10 Janssen Research and Development, Division of Janssen Pharmaceutica NV, Beerse, Belgium; 11 Department of Neurosciences, Faculty of Medicine, KU Leuven, Louvain, Belgium; 12 Laboratory of Cognitive Neurology, Department of Neurology, University Hospitals Leuven, Louvain, Belgium

**Keywords:** Alzheimer’s disease (AD), Amyloid-β 1–43 (Aβ_1–43_), Cerebrospinal fluid (CSF), Alzheimer mutations, Oxford Nanopore Technologies (ONT) long-read sequencing

## Abstract

**Background:**

Alzheimer’s disease (AD) mutations in amyloid precursor protein (*APP*) and presenilins (*PSENs*) could potentially lead to the production of longer amyloidogenic Aβ peptides. Amongst these, Aβ_1–43_ is more prone to aggregation and has higher toxic properties than the long-known Aβ_1–42_. However, a direct effect on Aβ_1–43_ in biomaterials of individuals carrying genetic mutations in the known AD genes is yet to be determined.

**Methods:**

*N* = 1431 AD patients (*n* = 280 early-onset (EO) and *n* = 1151 late-onset (LO) AD) and 809 control individuals were genetically screened for *APP* and *PSENs*. For the first time, Aβ_1–43_ levels were analysed in cerebrospinal fluid (CSF) of 38 individuals carrying pathogenic or unclear rare mutations or the common *PSEN1* p.E318G variant and compared with Aβ_1–42_ and Aβ_1–40_ CSF levels.

The soluble sAPPα and sAPPβ species were also measured for the first time in mutation carriers.

**Results:**

A known pathogenic mutation was identified in 5.7% of EOAD patients (4.6% *PSEN1*, 1.07% *APP*) and in 0.3% of LOAD patients. Furthermore, 12 known variants with unclear pathogenicity and 11 novel were identified. Pathogenic and unclear mutation carriers showed a significant reduction in CSF Aβ_1–43_ levels compared to controls (*p* = 0.037; < 0.001). CSF Aβ_1–43_ levels positively correlated with CSF Aβ_1–42_ in both pathogenic and unclear carriers and controls (all *p* < 0.001). The p.E318G carriers showed reduced Aβ_1–43_ levels (*p* < 0.001), though genetic association with AD was not detected. sAPPα and sAPPβ CSF levels were significantly reduced in the group of unclear (*p* = 0.006; 0.005) and p.E318G carriers (*p* = 0.004; 0.039), suggesting their possible involvement in AD. Finally, using Aβ_1–43_ and Aβ_1–42_ levels, we could re-classify as “likely pathogenic” 3 of the unclear mutations.

**Conclusion:**

This is the first time that Aβ_1–43_ levels were analysed in CSF of AD patients with genetic mutations in the AD causal genes. The observed reduction of Aβ_1–43_ in *APP* and *PSENs* carriers highlights the pathogenic role of longer Aβ peptides in AD pathogenesis. Alterations in Aβ_1–43_ could prove useful in understanding the pathogenicity of unclear *APP* and *PSENs* variants, a critical step towards a more efficient genetic counselling.

## Background

AD is the most common cause of dementia, characterized by progressive cognitive decline and memory loss, accounting for 50 to 75% of all dementia patients [1]. Based on the disease onset, AD is classified into early-onset AD (EOAD, < 65 years) and late-onset AD (LOAD, > 65 years) [2]. Mutations in amyloid precursor protein (*APP*), presenilin 1 (*PSEN1*) and presenilin 2 (*PSEN2*) have been identified as a cause of both EOAD and LOAD [3, 4] explaining 10% of all EOAD and about 2% of LOAD patients [4, 5]. The overproduction and aggregation of amyloid-β (Aβ) in the brain are thought to be the major causal events triggering AD that ultimately lead to neuronal loss [6]. Aβ peptides aggregate in the brain forming amyloid plaques, which together with neurofibrillary tangles (NFTs) of hyper-phosphorylated tau protein are the AD pathological hallmarks [6, 7]. Aβ is generated from cleavages of APP through at least two distinct and mutually exclusive pathways. In the so-called non-amyloidogenic pathway, APP is cleaved by α-secretase and γ-secretase to produce three fragments: a secreted C-terminal fragment (sAPPα), p3 and the APP intracellular domain (AICD) [4]. In the amyloidogenic (pathogenic) pathway, APP is cleaved by β-secretase, followed by γ-secretase cleavage. The cleavage by β-secretase generates a large soluble extracellular secreted domain (sAPPβ) and C99. The latter undergoes additional cleavages by γ-secretase to generate a series of Aβ peptides 39–43 amino acids long, following two different pathways: Aβ_1–49_ > Aβ_1–46_ > Aβ_1–43_ > Aβ_1–40_ and Aβ_1–48_ > Aβ_1–45_ > Aβ_1–42_ > Aβ_1–38_ [8]. Shorter peptides can also be produced, including Aβ_1–41_ from Aβ_1–43_ [9] and Aβ_1–34_ from either Aβ_1–42_ or Aβ_1–40_ [10], which is considered a biomarker of Aβ clearance and AD progression [11]. Pathogenic mutations in *APP* and *PSEN1* and *2* (γ-secretase’s catalytic subunits) are known to influence APP metabolism leading to the deposition of Aβ peptides. The most abundant Aβ peptides in the cerebrospinal fluid (CSF) resulting from APP processing are Aβ_1–38_, Aβ_1–40_ and Aβ_1–42_. The latter is considered the most pathological peptide in AD as it is most prone to aggregation into amyloid plaques [12]. Aβ_1–42_ levels have been found to be reduced in CSF of patients with AD [13] as a result of Aβ_1–42_ increased production and subsequent accumulation into plaques [14]. Furthermore, CSF Aβ_1–42_ levels are usually reduced up to decades before the clinical symptoms of dementia appear [15]. Thus, CSF Aβ_1–42_ levels are considered a core biomarker for early AD [16], together with total tau protein (T-tau) (non-AD specific) and tau phosphorylated at threonine 181 (P-tau181) (AD specific) [17]. In addition to Aβ peptides, α and β cleaved soluble APP (sAPPα and sAPPβ) are products of the APP metabolism which also have been investigated as possible AD biomarkers, though with contradicting results [18, 19]. Aβ_1–42_ levels have been used for the pathological classification of AD mutations, both in vivo and in vitro [20]. However, some of the established AD pathogenic mutations do not show altered Aβ_1–42_ levels [21]. For example, in the brain of the *PSEN1* p.R278I knock-in mice, a decrease of Aβ_1–40_ was accompanied by an increase of another Aβ, i.e. species, Aβ_1–43_, which showed higher aggregative properties than Aβ_1–42_ [21]. Interestingly, Aβ_1–43_ was also detected in the brain of sporadic and familial AD patients [22–24] supporting the hypothesis that the generation of relatively long Aβ peptides (>Aβ_1–42_) could explain part of the pathogenic effect of the known deleterious *PSENs* and *APP* mutations [25, 26]. Furthermore, several variants identified in the causal AD genes remain of uncertain significance (VUS) (www.alzforum.org/mutations; AD/FTD Mutation Database [27]), due to lack of functional studies and co-segregation with disease in relatives. Understanding the role of these variants is important for a correct clinical diagnosis, for genetic counselling and for the selection of well-stratified patient groups for clinical trials. The investigation of longer Aβ peptides, including Aβ_1–43_, in CSF of individuals carrying AD mutations or VUS could disclose possible alterations of the Aβ peptide production and explain their possible role in the AD pathogenesis. Recent studies showed that CSF Aβ_1–43_ levels are significantly reduced in individuals with AD and mild cognitive impairment (MCI) [16, 28], as well as in EOAD patients compared to LOAD [29], independently from the mutation status. Studies assessing Aβ_1–43_ levels in CSF of *PSENs* and *APP* mutation carriers are therefore lacking. In the present work, we measured for the first time the Aβ_1–43_ levels in CSF of carriers of *APP* and *PSENs* pathogenic and VUS mutations and control individuals and we compared these results to Aβ_1–42_ and Aβ_1–40_. We also measured, for the first time in mutation carriers, sAPPα and sAPPβ in relation to Aβ_1–43_, as they are less investigated, but still part of the APP processing.

## Methods

### Study population

The study population consisted of 1431 AD patients (62.03% [*n* = 889] women, average age at onset (AAO) 73.51 ± 9.81 years, range 29–96 years), ascertained in Belgium through the neurology centres of the clinical partners of the Belgian neurology (BELNEU) consortium. All patients received a diagnosis of possible, probable or definite AD according to the criteria described by the National Institute of Neurological and Communication Disorders and Stroke Alzheimer’s Disease and Related Disorders Association (NINCDS-ADRDA) criteria and the National Institute on Aging–Alzheimer’s Association (NIA-AA) [30]. A positive family history of dementia (i.e. at least one first-degree relative affected) was documented in 22.22% (*n* = 318) of the patients. Based on the onset age (< 65 years), 19.57% (*n* = 280) of the patients were classified as having early-onset AD (EOAD, 54.64% [*n* = 153] women, average AAO 58.22 ± 6.14, age range 29–65 years). A Belgian control cohort of 809 individuals (73.54% [*n* = 595] women, average age at inclusion (AAI) 70.12 ± 10.71, 31–96 years) was included in the study. The control individuals were primarily community-dwelling volunteers or spouses of patients. Subjective memory complaints, neurologic or psychiatric antecedents, and a familial history of neurodegeneration were ruled out by means of an interview. Cognitive screening was performed using the Mini-Mental State Examination (cut-off score > 26) [31] and/or the Montreal Cognitive Assessment test (cut-off score > 25) [32]. The spouses of patients were examined at the Memory Clinic of ZNA Middelheim and Hoge Beuken in Antwerp, Belgium. CSF of additional 64 controls individuals (AAI 68.05 ± 5.97), used in this study for comparison purposes, were available at the Reference Centre for Biological Markers of Dementia (BIODEM), Department of Biomedical Sciences, University of Antwerp. These were primarily community-dwelling volunteers enrolled in the BACEi program (ClinicalTrials.gov identifiers: 54861911ALZ1005 (NCT01978548); 54861911ALZ2002 (NCT02260674)) by Janssen Pharmaceutica NV, Beerse, Belgium [33]. Subjective memory complaints, neurologic or psychiatric antecedents, were ruled out by means of an interview. Cognitive screening was performed using the mini-mental state examination (cut-off score > 26). The Clinical Dementia Rating Scale (CDR) score was 0, and AD CSF biomarkers were within the normal range. Genetic screening in these 64 individuals was not performed due to the unavailability of DNA. All research participants or their legal guardian provided written informed consent for participation in genetic and clinical studies. Clinical study protocols and informed consent forms for patient ascertainment were approved by the local medical ethics committees of the collaborating medical centres in Belgium. Genetic study protocols and informed consent forms were approved by the ethics committees of the University Hospital of Antwerp and the University of Antwerp, Belgium.

### Mutation screening

Genomic DNA of patients and control individuals was analysed for mutations in *APP*, *PSEN1* and *PSEN2* using a targeted re-sequencing gene-panel as we previously described [34]. We selected non-synonymous variants with a minor allele frequency (MAF) < 1%, including exon-intron boundaries for possible variants affecting splicing. The allelic frequencies of these variants, observed in the patient group, were assessed in the control cohort and Genome Aggregation database (gnomAD, lastly accessed in January 2020) [35]. In addition, *PSEN1* rs17125721 A > G, p.E318G variant (MAF 1.927%, gnomAD), considered a possible risk modifier (https://www.alzforum.org/mutations/psen1-e318g) and Apolipoprotein E (*APOE*) genotypes were analysed. Variants with a read depth below 20X, with Genoqual value below 99 and with an imbalanced wild-type/variant read depth (cut-off > 3) were considered false positives. All the selected variants were validated by Sanger sequencing (BigDye Terminator Cycle sequencing kit v3.1) on the ABI 3730 DNA Analyser (both Applied Biosystems). Sequences were analysed using SeqManII or novoSNP software packages [36]. To screen for *APP* locus duplications, multiplex amplicon quantification (MAQ) was used on 280 EOAD patients, as previously described [37]. Briefly, multiplex PCR amplification of 5 target and 11 reference amplicons was performed using fluorescently labelled primers. The amplification products were size separated on ABI 3730 automatic sequencer using GeneScan-500 LIZ (Applied Biosystems) as internal size standard. Data analysis was performed using the MAQ software (MAQs) package (www.vibgeneticservicefacility.be). One sample carrying the *APP* duplication and four different control samples were used as positive and negative controls, respectively.

### CSF biomarker analysis

CSF were available for a subset of 38 mutation carriers: 18 carriers of pathogenic and VUS mutations (MAF < 1%) (AAO 71.58 ± 11.24 years, range 56–85 years) and 20 carriers of *PSEN1* p.E318G variant (AAO 78.09 ± 6.62 years, range 62–87 years) as well as 64 control individuals (AAI 68.05 ± 5.97, cfr. “Study population”). Lumbar puncture, CSF sampling and handling have been performed according to standard protocols [12]. Samples were stored at − 80 °C until analysis. CSF concentrations of Aβ_1–42_ and Aβ_1–40_ were measured using enzyme-linked immunosorbent assay (ELISA) with commercially available single parameter ELISA kits at the BIODEM laboratory as previously described [38]. Aβ_1–43_ levels were measured using the amyloid beta (1–43) (FL) Aβ kit (IBL) and both sAPPα and sAPPβ using sAPPα/sAPPβ kit (Meso Scale Discovery). Concerning T-tau and P-tau181 levels, these were previously measured using hTAU Ag and PHOSPHO-TAU(181P) (INNOTEST), respectively, and the BIODEM laboratory performed the ELISA for the 38 mutation carriers and the Sahlgrenska University Hospital (Sweden) for the 64 control individuals. For this reason, we did not perform statistical analysis on either T-tau or P-tau181 amongst the groups, but we only reported the values of the measurements, which are part of the phenotypic characterization of the cohort. All calibration standards and CSF samples were analysed in duplicate. Only mean values with a coefficient of variation (CV) of the replicates less than or equal to 20% were included in the analysis.

### Statistical analyses

Statistical analyses were performed in SPSS version 24, and graphs were made using GraphPad Prism8. A logistic regression analysis was performed in the AD and control cohorts, to determine whether patients had higher probability than controls to carry both the common *PSEN1* p.E318G and *APOE* ε4 genotype. With a similar model, we further tested the prevalence of *APOE* ε4 between EOAD and LOAD cohorts and of *APOE* ε4ε4 between EOAD and LOAD cohorts.

For the CSF biomarker analysis, the mutation carriers were divided in three different groups: carriers of known pathogenic mutations, of VUS and of *PSEN1* p.E318G. First, a Kolmogorov-Smirnov test was performed to check for normal distribution. Since most variables did not follow a normal distribution, non-parametric tests were used. Differences amongst groups were tested using Kruskal-Wallis. Post hoc analysis with pairwise comparisons was carried out (adjusted *p* value < 0.05). To identify whether an association between two markers was present, the Spearman’s rho correlation tests were performed. Correlation coefficients were extracted from each group (controls, known pathogenic, VUS, *PSEN1* p.E318G) separately. After correction for multiple testing (Bonferroni’s correction), *p* values < 0.005 or below were considered to be statistically significant. Receiver operating characteristic (ROC) curves were additionally performed to assess the diagnostic accuracy of the individual biomarkers, and the area under each ROC curve (AUC) was calculated. Finally, explanatory cut-offs were identified as the concentration of specific biomarker that maximizes sensitivity and specificity of the test (Youden’s index).

### Transcript analysis

To inhibit non-sense-mediated mRNA decay (NMD), 1 × 10^6^ lymphoblast cells of the double mutation carrier (patient 16; *PSEN1* p.G183V, *PSEN1* p.P49L), of the single carrier (sibling of patient 16; *PSEN1* p.G183V) and of 4 non-carriers as negative controls were incubated with 150 mg/mL cycloheximide (CHX) (Sigma-Aldrich) at 37 °C for 4 h. To generate cDNA, total RNA was extracted using the RiboPureTM kit (Life Technologies) followed by a DNase treatment (TURBODNase Kit, Invitrogen). First-strand complementary DNA was synthesized using the SuperScript III First-Strand Synthesis System (Life Technologies) using random hexamer primers. Full-length *PSEN1 t*ranscript sequencing was subsequently performed on a MinION sequencing platform (Oxford Nanopore Technologies (ONT)) based on a modernized cDNA sequencing protocol [39]. Briefly, cDNA amplification of *PSEN1* full transcript was carried out with Platinum® Taq DNA Polymerase (Clontech Laboratories) using exonic primers, designed with Primer3Plus [40], with a 5′ ONT adapter. In a second PCR round, using PCR Barcoding Kit 96 (EXP-PBC096; ONT), sample-specific barcodes were added. Samples were pooled equimolar and processed according to ONT SQK-LSK109 library preparation. Sequencing was performed on a Mk1 MinION (MIN-101B), using FLO-MIN106 flow cells. Base calling and barcode de-multiplexing were performed with Albacore (v2.2.5). Sequencing reads were subsequently aligned using minimap2 [41], with splice-aware parameters. Only full-length *PSEN1* spanning sequencing reads were retained for further analysis. Relative quantifications of splice junctions were calculated by dividing the number of junction-supporting reads by the total number of reads spanning the *PSEN1* transcript in R (R Core Team, 2017).

## Results

### Mutation screening

The genetic screening identified a total of 41 mutations in 54 individuals (41/1431, 2.86% AD, 13/809, 1.60% controls). The identified variants are listed in Table S1 (Additional file). Fourteen were known pathogenic mutations (4 in *APP*, 10 in *PSEN1*) (www.alzforum.org/mutations, lastly accessed in January 2020) and were identified in 19 patients (19/1431, 1.33%) and 1 control individual (1/809, 0.12%). Specifically, 1.07% (3/280) of EOAD patients carried known pathogenic mutations in *APP* and 4.6% (13/280) in *PSEN1.* Furthermore, in 0.086% (1/1151) and 0.26% (3/1151) of LOAD patients, known pathogenic mutations were identified in *APP* and *PSEN1*, respectively.

In addition, 12 previously reported variants with unclear pathogenicity (5 in *APP*, 2 *PSEN1*, 5 *PSEN2*) were detected in 12 patients (12/1431, 0.84%) and 7 controls (7/809, 0.86%). Three were known benign variants (2 *APP*, 1 *PSEN2*) found in 7 patients (7/1431, 0.49%) and 2 controls (2/809, 0.24%). Lastly, 11 rare variants were novel (5 in *APP*, 1 in *PSEN1*, 5 in *PSEN2*) absent from gnomAD and mutation databases, observed in 8 patients (8/1431, 0.56%) and 3 control individuals (4/809, 0.49%). There was no significant difference between the prevalence of *APOE* ε4 in EOAD (57%) and LOAD (55%) (*p* = 0.48). The prevalence of *APOE* ε4 was higher in EOAD (60%) than LOAD (55%) only on individuals without any *APP* or *PSENs* mutations, but this difference was still not significant (*p* = 0.15). We found a significant difference in the prevalence of *APOE* ε4ε4 between EOAD (20%) and LOAD (9%) when considering all individuals (*p* < 0.001). This difference in the prevalence of *APOE* ε4ε4 (EOAD 20%; LOAD 9%) was similar when running the same model only on individuals without any *APP* or *PSENs* mutations (*p* < 0.001).

*APOE* genotypes of the mutation carriers are listed in Table S1 (Additional file). Amongst the patients, two were double mutation carriers: patient 16, carrying both *PSEN1* p.G183V and *PSEN1* p.P49L, with LOAD pathology (Figure S1, Additional file), and patient 11, carrying both *APP* p.G625_S628del and *PSEN1* p.P355S. The risk variant *PSEN1* p.E318G was detected in 45 patients (45/1431, 3.14%) and 24 controls (24/809, 2.96%). No significant association was found between *PSEN1* p.E318G and AD, regardless of *APOE* ε4 genotype (*p* = 0.29 and *p* = 0.51, respectively). Furthermore, in the screened cohort, *APP* locus duplications were not detected.

### CSF biomarker analysis

**Fig. 1:**
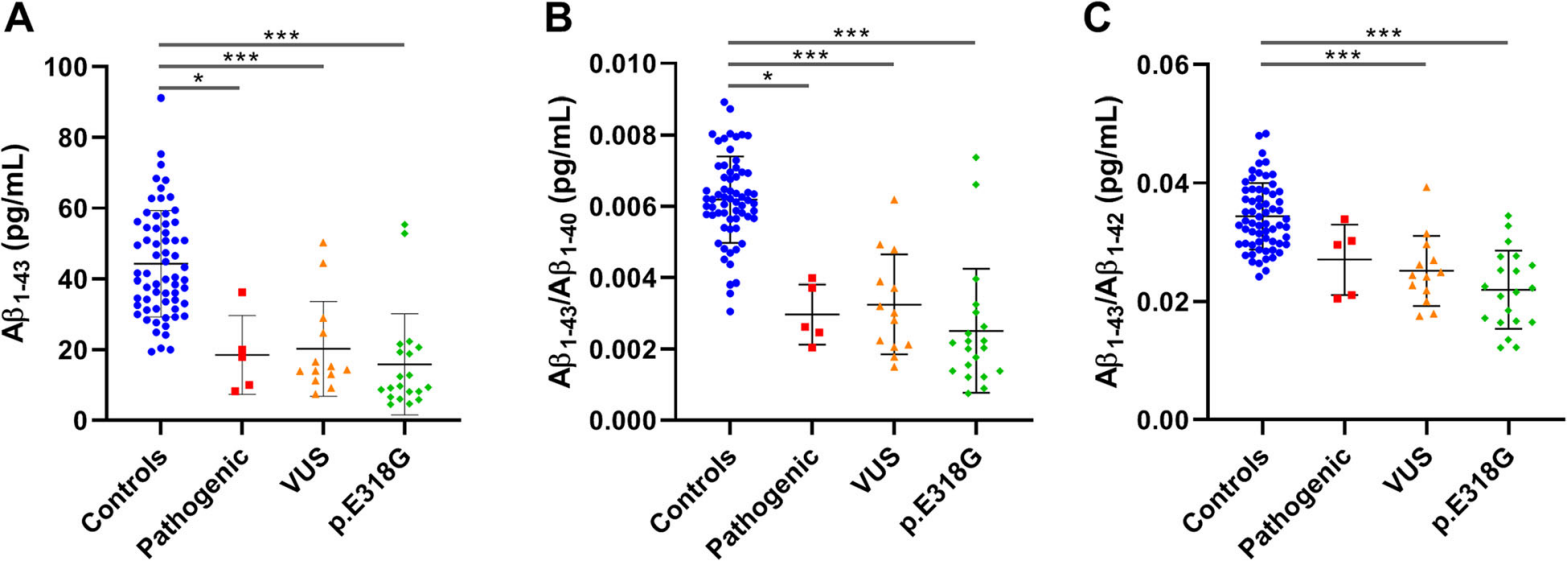
CSF levels of Aβ_1–43_, Aβ_1–43_/Aβ_1–40_ and Aβ_1–43_/Aβ_1–42_ ratios in the three mutation carrier groups compared to controls. Scatter plots show Aβ_1–43_ (**a**), Aβ_1–43_/Aβ_1–40_ (**b**) and Aβ_1–43_/Aβ_1–42_ (**c**) CSF levels in controls and in carriers of known pathogenic mutations, of VUS and of *PSEN1* p.E318G. Values of mean ± SD are given. *p* value indicators correspond to the values assessed with Kruskal-Wallis: *p < 0.05, ***p < 0.0001

**Fig. 2:**
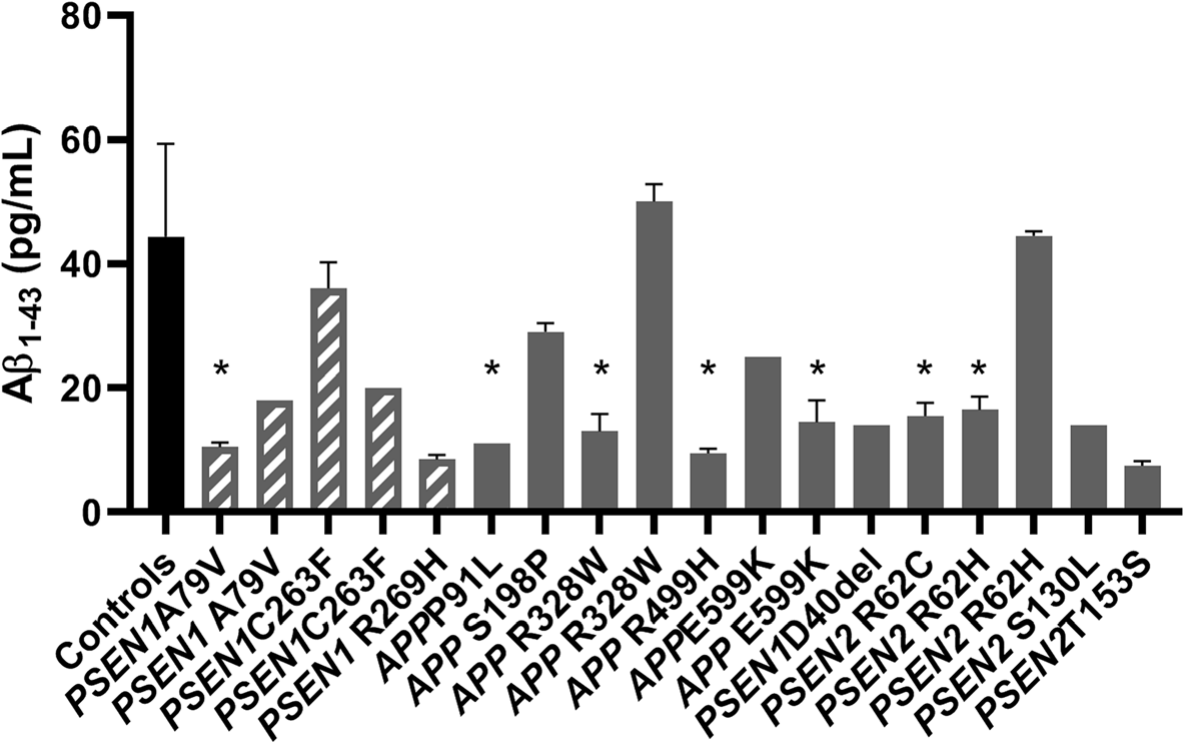
Aβ_1–43_ CSF levels in carriers of known pathogenic mutations or VUS compared with the control group. The CSF levels of Aβ_1–43_ for each carrier of a known pathogenic mutation (in stripes) or a VUS (in gray) are shown in the bar plots together with the control group (in black). Error bars indicate the SD of the duplicate measurements for the mutation carriers and the average of the values for the controls. The asterisks (*) indicate the carriers of one *APOE* ε4 allele

**Fig. 3:**
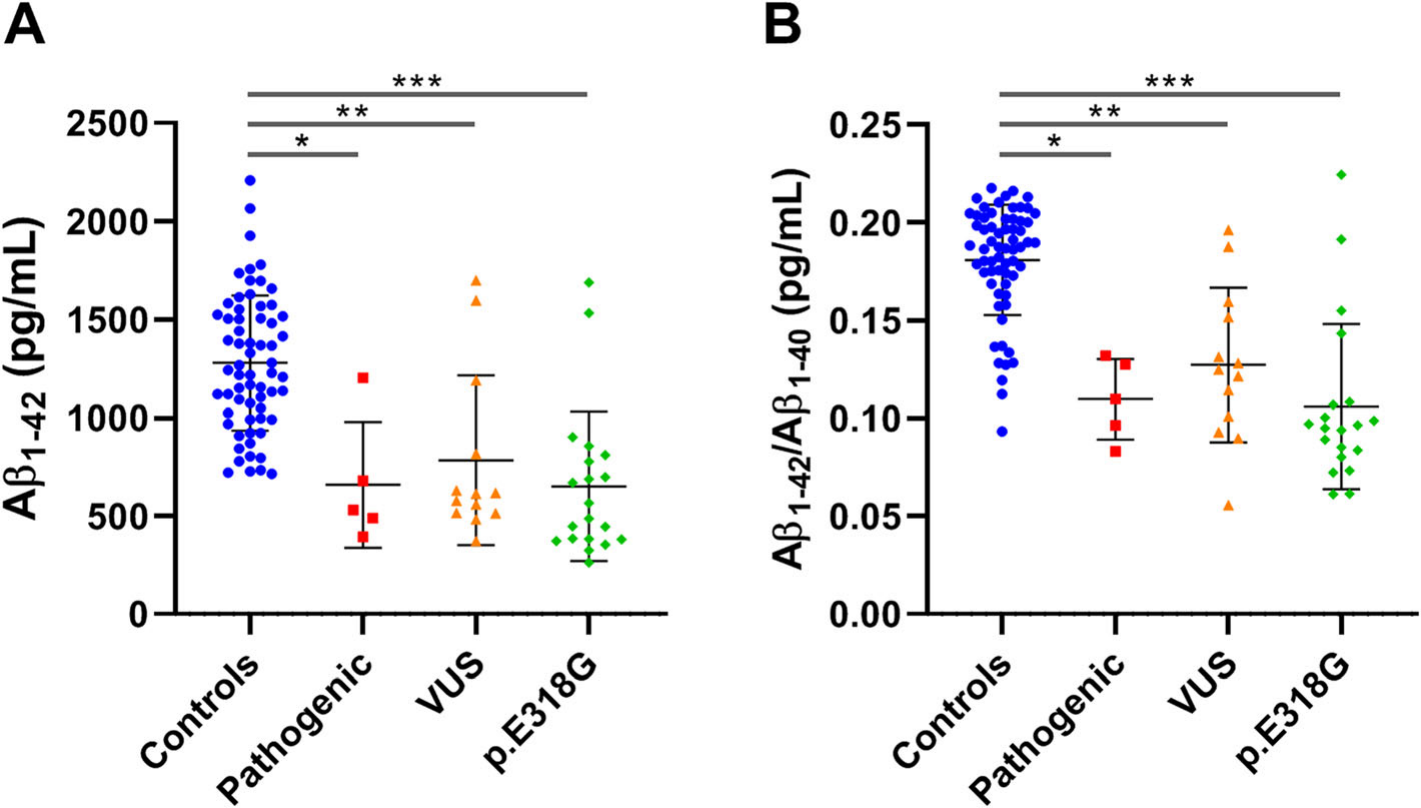
CSF levels of Aβ_1–42_ and Aβ_1–42_/Aβ_1–40_ ratio in the three mutation carrier groups compared to controls. Scatter plots show Aβ_1–42_ (**a**) and Aβ_1–42_/Aβ_1–40_ (**b**) CSF levels in controls and in carriers of known pathogenic mutations, of VUS and of *PSEN1* p.E318G. Values of mean ± SD are given. *p* value indicators correspond to the values assessed with Kruskal-Wallis: *p < 0.05, **p < 0.01, ***p < 0.0001

The CSF biomarkers were analysed comparing three groups of mutation carriers: (1) known pathogenic, (2) VUS and (3) *PSEN1* p.E318G to control individuals. A significant reduction of CSF Aβ_1–43_ levels was detected in all three carrier groups (all *p* < 0.05; Table 1; Fig. 1). For each carrier of a known pathogenic mutation or a VUS, Aβ_1–43_ levels are shown in the bar plot (Fig. 2). Similarly, Aβ_1–42_ CSF levels were significantly reduced in all three carrier groups (all *p* < 0.05; Table 1; Fig. 3). Comparison of CSF Aβ_1–40_ levels did not show a significant difference amongst all groups (*p* = 0.66).

**Table 1:** Significance levels (adjusted *p* values) of the markers compared amongst groups

	***Pathogenic*** vs ***controls***			***VUS*** vs ***controls***			***p.E318G*** vs ***controls***		
	Est.	*St.*	*p* value	Est.	*St.*	*p* value	Est.	*St.*	*p* value
Aβ_1–43_	39.93	2.90	**0.037**	37.36	4.15	**< 0.001**	45.43	5.99	**< 0.001**
Aβ_1–43_/Aβ_1–40_	42.92	3.12	**0.018**	40.08	4.45	**< 0.001**	46.97	6.20	**< 0.001**
Aβ_1–43_/Aβ_1–42_	28.45	2.07	0.384	37.37	4.15	**< 0.001**	44.85	5.91	**< 0.001**
Aβ_1–42_	40.32	2.93	**0.033**	32.33	3.59	**0.003**	41.42	5.46	**< 0.001**
Aβ_1–42_/Aβ_1–40_	43.52	3.14	**0.015**	33.59	3.73	**0.002**	43.87	5.79	**< 0.001**
sAPPα	12.54	0.91	1.0	31.04	3.45	**0.006**	26.90	3.55	**0.004**
sAPPβ	14.06	1.02	1.0	31.32	3.48	**0.005**	21.87	2.89	**0.039**
Aβ_1–43_/sAPPα	37.44	2.73	0.064	21.18	2.35	0.186	38.79	5.12	**< 0.001**
Aβ_1–43_/sAPPβ	36.31	2.64	0.082	21.49	2.39	0.169	41.25	5.44	**< 0.001**

Kruskal-Wallis was performed to assess differences amongst groups. Significance level *p* < 0.05 (adjusted *p* values). *Abbreviations*: *Est.* test estimate, S*t.* standard test statistic

The Aβ_1–43_/Aβ_1–40_ and Aβ_1–42_/Aβ_1–40_ ratios were significantly reduced in all three carrier groups (all *p* < 0.05, Table 1; Figs. 1, 2 and 3). Aβ_1–43_/Aβ_1–42_ ratio was significantly reduced in the VUS and *PSEN1* p.E318G carrier groups (both *p* < 0.001) (Fig. 1). Aβ_1–43_ significantly correlated with Aβ_1–42_ in all four groups (*r* > 0.87, all *p* < 0.001). Aβ_1–43_ and Aβ_1–42_ were significantly correlated with Aβ_1–40_ in the controls and *PSEN1* p.E318G group (all *r* > 0.63, all *p* < 0.001), while there was a correlation between Aβ_1–42_ with Aβ_1–40_ in the VUS group, but not significant after Bonferroni’s correction (*r* = 0.65, *p* = 0.015). Both CSF Aβ_1–43_ and Aβ_1–42_ were able to differentiate the corresponding controls from the group of known pathogenic and *PSEN1* p.E318G (AUCs > 0.9; Table S2, Additional file), while in the VUS group the differentiation was slightly less efficient (AUCs 0.84–0.89; Table S2, Additional file).

**Fig. 4:**
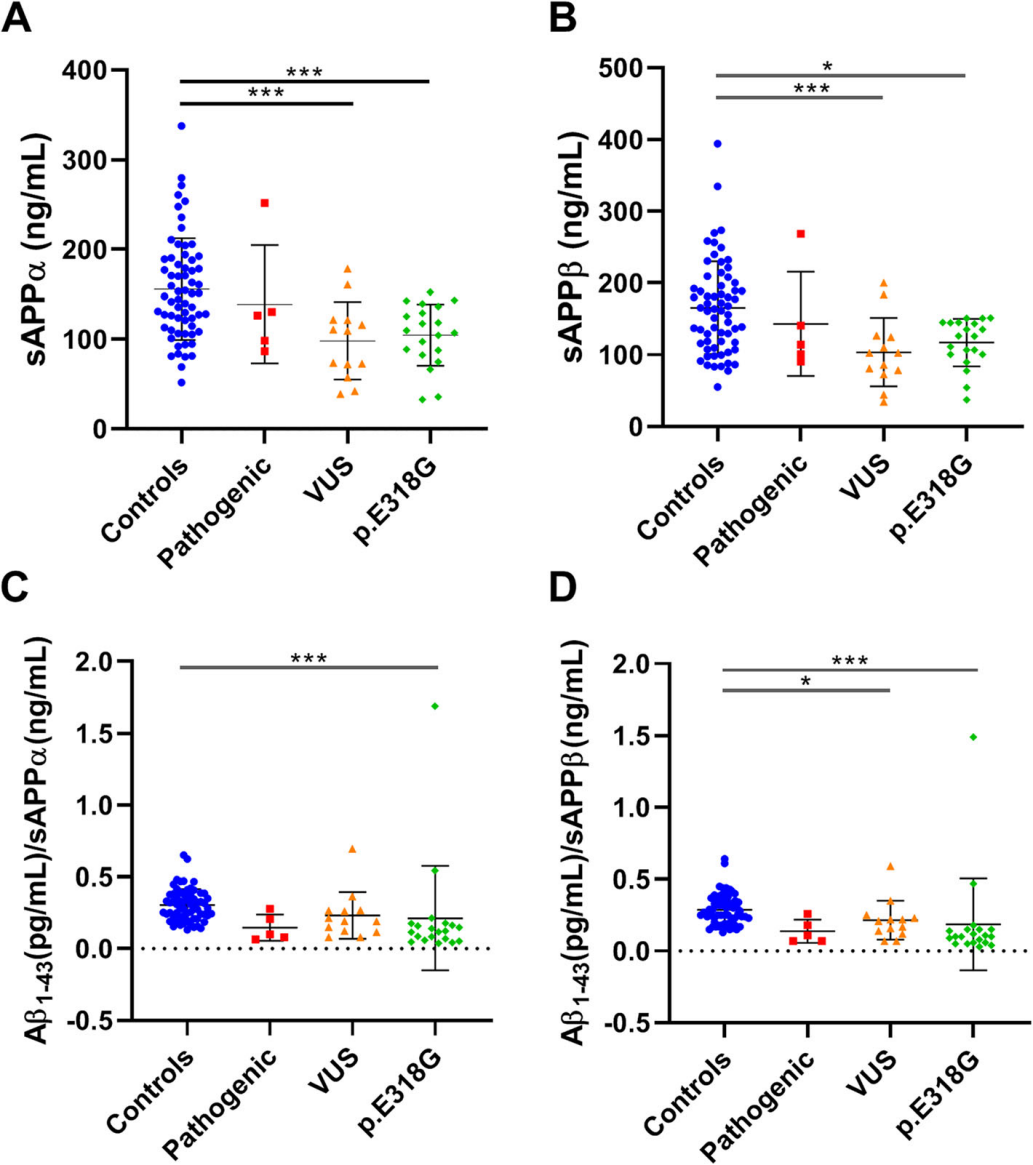
CSF levels of sAPPα, sAPPβ, Aβ_1–43_/sAPPα and Aβ_1–43_/sAPPβ ratios in the three mutation carrier groups compared to controls. Scatter plots show sAPPα (**a**), sAPPβ (**b**), Aβ_1–43_/sAPPα (**c**) and Aβ_1–43_/sAPPβ (**d**) CSF levels in controls and in carriers of known pathogenic mutations, of VUS and of *PSEN*1 p.E318G. Values of mean ± SD are given. *p* value indicators correspond to the values assessed with Kruskal-Wallis: *p < 0.05, ***p < 0.0001

CSF sAPPα and sAPPβ levels were significantly reduced in both VUS (both *p* < 0.05) and *PSEN1* p.E318G carriers (both *p* < 0.04) (Table 1; Fig. 4).

Aβ_1–43_ and Aβ_1–42_ showed a positive correlation with both sAPPα and sAPPβ only in the control group (all *r* > 0.48, all *p* < 0.001). Aβ_1–40_ correlated with sAPPα only in the control group (*r* = 0.6, *p* < 0.001) and with sAPPβ in the control (*r* = 0.65, *p* < 0.001) and VUS groups (*r* = 0.75, *p* = 0.003). Aβ_1–43_/sAPPα and Aβ_1–43_/sAPPβ CSF levels were significantly lower in *PSEN1* p.E318G carriers compared to controls (*p* < 0.001) (Fig. 4). Aβ_1–43_/sAPPα and Aβ_1–43_/sAPPβ CSF levels also showed a trend to be decreased in carriers of the pathogenic mutations, although this difference was not statistically significant (*p* = 0.06 and *p* = 0.08, respectively) (Table 1; Fig. 4). The AUCs for both sAPPα and sAPPβ were low (AUCs = 0.625; Table S2, Additional file). CSF levels for each marker (Aβ_1–43_, Aβ_1–42_, Aβ_1–40_, sAPPα, sAPPβ) are summarised in Table 2. ROC curve analysis for all biomarkers are listed in Table S2 (Additional file), and the AUC curves are shown in Figures S2-S3 (Additional file).

**Table 2:** Summary of CSF marker levels for each mutation carrier

	Individual identifier	Mutation	***APOE***	Aβ_**1–43**_	Aβ_**1–42**_	Aβ_**1–40**_	sAPPα	sAPPβ	T-tau	P-tau181
P	**Patient 12**	*PSEN1* p.A79V	34	**10**	**490**	**3836**	**99**	**90**	NA	NA
	**Patient 13**	*PSEN1* p.A79V	33	**18**	**531**	**4833**	**87**	**101**	959	136
	Patient 17	*PSEN1* p.C263F	33	**20**	**679**	8165	252	268	458	73
	**Patient 21**	*PSEN1* p.C263F	33	36	1202	9117	**130**	**141**	281	55
	**Patient 24**	*PSEN1* p.R269H	33	**8**	**393**	**4063**	**127**	**114**	640	77
V	Patient 10	*APP* p.P91L	34	**11**	**516**	**4026**	**42**	**44**	287	44
	Control 3	*APP* p.S198P	33	29	1190	7838	**111**	**125**	248	47
	Patient 5	*APP* p.R328W	33	50	1598	8134	**72**	**86**	371	76
	Control 4	*APP* p.R328W	34	**13**	**483**	8679	161	184	NA	NA
	Patient 6	*APP* p.R499H	34	**9**	**513**	**4486**	**74**	**78**	440	51
	Patient 7	*APP* p.E599K	34	**14**	**816**	8065	179	200	> 1200	225
	Patient 8	*APP* p.E599K	33	25	**630**	5184	**116**	**110**	633	104
	Patient 29	*PSEN1* p.D40del	33	**14**	**618**	6641	**122**	**103**	615	82
	Patient 31	*PSEN2* p.R62C	34	**15**	**613**	6823	**57**	**73**	NA	NA
	Patient 32	*PSEN2* p.R62H	33	44	1699	9048	**122**	**127**	407	59
	Patient 34	*PSEN2* p.R62H	34	**17**	**561**	**4269**	**110**	**103**	783	82
	Patient 36	*PSEN2* p.S130L	33	**14**	**578**	**4631**	**73**	**81**	340	54
	**Patient 40**	*PSEN2* p.T153S	33	**7**	**373**	**2340**	**39**	**34**	212	32

List of carriers of *APP* and *PSENs* known pathogenic (P) and VUS (V) mutations with CSF available. Notes: all carriers had AD diagnosis except control 3 and control 4, who were controls at the moment of inclusion. Control 4 developed vascular dementia at follow-up (AAO 75 years). Patients in bold are EOAD patients (AAO range 56–65 years). Values for Aβ_1–43_, T-tau and P-tau181 are in pg/mL. Underlined values and values in bold, for Aβ_1–43_, Aβ_1–42_, Aβ_1–40_, sAPPα and sAPPβ, are respectively higher and lower levels of the markers based on the exploratory cut-offs (Table S2, Additional file). Normal cut-offs: T-tau < 297 pg/mL; P-tau181 < 57 pg/mL

### PSEN1 full transcript analysis

**Fig. 5:**
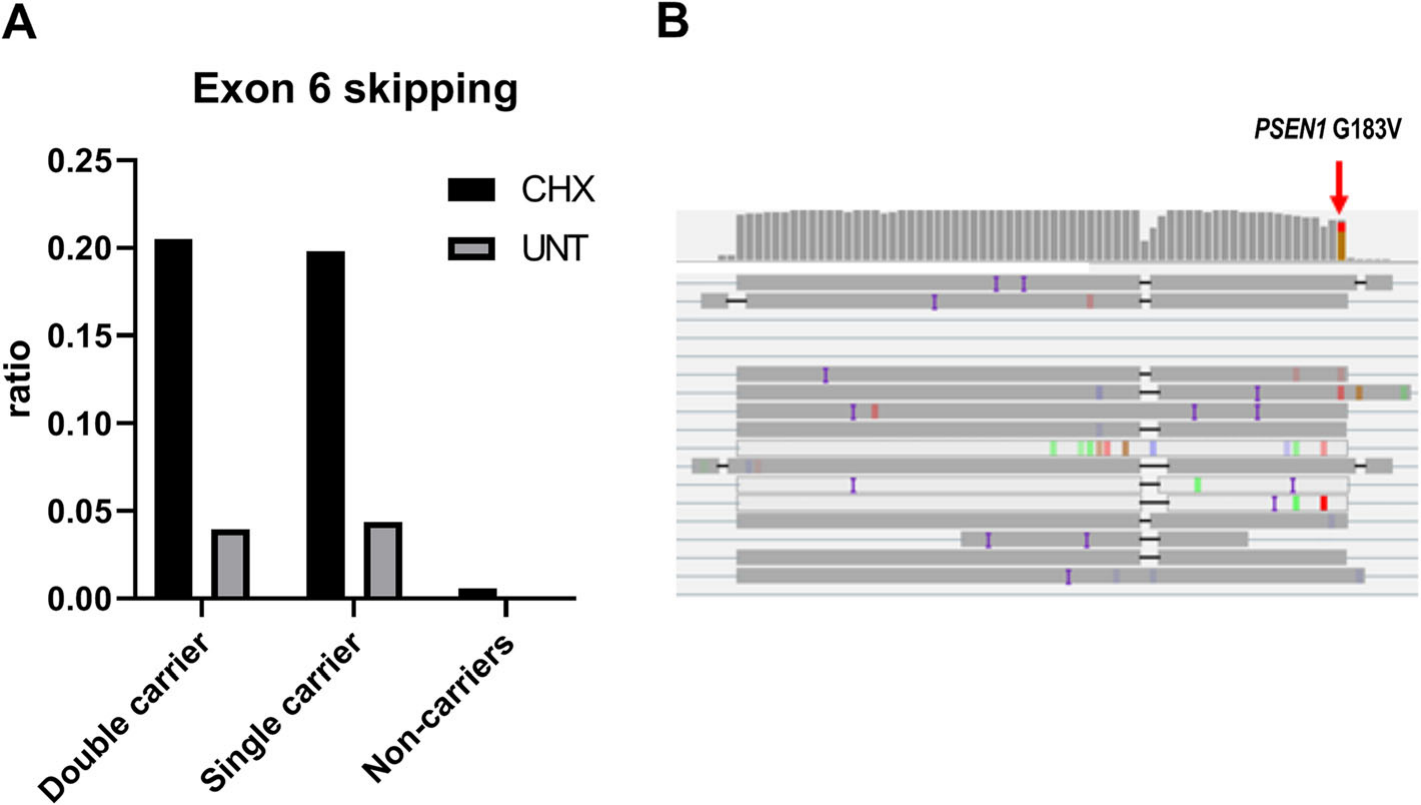
Transcript analysis of PSEN1 in patient 16. The bar graph shows the relative quantifications of exon 6 in the double carrier (patient 16, *PSEN1* p.G183V, p.P49L), single carrier (*PSEN1* p.G183V) and non-carrier lymphoblast cells CHX treated (CHX) and untreated (UNT). Relative quantifications of splice junctions were calculated by dividing the number of junction-supporting reads by the total number of reads spanning the PSEN1 transcript. The quantifications for both CHX and UNT of the non-carriers are reported as averages (values of SD for CHX ± 0.001052869 and for UNT ± 0.000671837) (**a**). Visualization of the PSEN1 exon 6 cDNA MinION reads from Integrative Genomic Viewer software (IGV). Sequencing reads of PSEN1 cDNA of the double and single carriers confirm exon 6 skipping due to the *PSEN1* p.G183V mutation (**b**)

The effect of the *PSEN1* p.G183V and *PSEN1* p.P49L double mutation on PSEN1 alternative transcript generation in patient-derived RNA was examined with ONT MinION sequencing, because of a previously reported effect of this mutation on exon skipping in both HEK293 cells [42] and mice brain [43]. Both the double mutation carrier (patient 16; *PSEN1* p.G183V, *PSEN1* p.P49L) and the single *PSEN1* p.G183V carrier show the skipping of exon 6 in 20% of the sequencing reads from the cDNA obtained from the lymphoblast cells treated with CHX and 5% from the untreated (Fig. 5). Exons 6–7 skipping was also detected, but it was a rarer event (Figure S4, Additional file). The analysis of the transcript surrounding the *PSEN1* p.P49L variants did not show splicing alterations (data not shown).

## Discussion

### Mutation screening

The *APP*, *PSEN1* and *PSEN2* mutation screening in 1431 AD patients and 809 control individuals identified known pathogenic mutations in 5.7% of EOAD (16/280) and 0.3% of LOAD (4/1151) patients and in 0.12% (1/809) of the controls, in line with what it is usually observed [4, 5]. The carrier of the *APP* p.A713T mutation considered with unclear pathogenicity but actually showing segregation with the disease in another study [44] was asymptomatic at the age of inclusion in our cohort (age > 65 years) [34]. This *APP* mutation is known to have a wide onset age range; therefore, it is not surprising to identify asymptomatic carriers [34, 44]. Amongst the known pathogenic mutations, two were also found in AD patients with more than 65 years of onset: the known pathogenic *PSEN1* p.C263F, identified in five patients with onset age range of 53–70, and the *PSEN1* p.G183V, previously identified in a patient with frontotemporal dementia and Pick-type tauopathy [42] (further discussed later in the text). These findings highlight the importance to screen the causal AD genes also in LOAD patients [5]. The genetic screening identified also two double mutation carriers: patient 11 carrying the *APP* p.G625_S628del and the *PSEN1* p.P355S, both VUS, and patient 16 having the *PSEN1* p.G183V and the novel p.P49L.

### *PSEN1* p.G183V and p.P49L mutation carrier

Patient 16 received a diagnosis of probable AD (age at onset > 65). Clinically, patient 16 showed amnestic presentation without other remarkable signs or symptoms. Single-photon emission computed tomography (SPECT) displayed a moderate hypoperfusion of the bilateral parietal, temporal and frontal lobe, compatible with AD. Magnetic resonance imaging (MRI) showed age-related atrophy and multiple supratentorial lacunary infarcts. Neuropathological examination showed a mild atrophy of the frontotemporal gyri (Figure S1, Additional file), but no FTD symptoms were reported in patient 16. The patient was pathologically diagnosed with definite AD (Montine stage A3B3C3), with an accent on the neurofibrillary pathology (Figure S1). The sibling of patient 16 also carried the *PSEN1* p.G183V, but not the *PSEN1* p.P49L, and was diagnosed with probable AD. Both *PSEN1* mutations therefore segregated independently in the family [45]. The *PSEN1* p.G183V was previously identified in a patient diagnosed with frontotemporal dementia (FTD) and Pick-type tauopathy [42]. The brain lesions in patient 16 were however different from those of the published FTD patient, where severe frontotemporal atrophy and Pick-like pathology were described [42]. There were no intranuclear neuronal inclusions in patient 16 and no signs of Pick’s disease. Thus, the Pick’s pathology detected in the FTD patient [42] is probably independent of this *PSEN1* p.G183V mutation. The *PSEN1* p.G183V is located in the last nucleotide of the exonic splice donor site of exon 6, and experiments in HEK293 cells and mice showed the formation of alternative transcripts with skipping of exons 6 and exon 6–7, which are likely degraded by the non-sense-mediated mRNA decay control mechanism (NMD) [42, 43]. Our PSEN1 transcript analysis with ONT minION sequencing, in patient-derived biomaterials, confirmed that *PSEN1* p.G183V led to the formation of transcripts lacking exons 6 and 6–7, which are indeed degraded by NMD. Only a small amount of these alternative transcripts remains in the cells (≤ 5%) and they unlikely interfere with the wild-type *PSEN1*. The presence of the second *PSEN1* mutation (p.P49L) could drive the disease in patient 16.

### Reduced Aβ_1–43_ CSF levels in *APP* and *PSENs* mutation carriers

CSF of 38 *APP*, *PSEN1* and *PSEN2* mutation carriers, both known pathogenic and VUS, was available and used to investigate for the first time the CSF Aβ_1–43_ levels. A significant reduction of CSF Aβ_1–43_ levels was observed in carriers of pathogenic mutations (13/18), of VUS (9/13) and of carriers of *PSEN1* p.E318G (18/20) compared to controls. This reduction was comparable with CSF Aβ_1–42_ levels. CSF Aβ_1–43_ levels positively correlated with Aβ_1–42_ in all mutation carrier groups but correlated with Aβ_1–40_ only in *PSEN1* p.E318G mutation carriers and controls. Aβ_1–43_/Aβ_1–40_ ratio was significantly reduced in all three mutation carrier groups, while Aβ_1–43_/Aβ_1–42_ ratio only in the VUS and *PSEN1* p.E318G groups, as previously shown for AD patients (independently from the mutation status) [9].

While longer Aβ peptides (e.g. Aβ_1–42_ and Aβ_1–43_) promote aggregation and neurotoxicity, Aβ_1–40_ appears to act protectively [46]. Moreover, CSF Aβ_1–40_ levels did not differ significantly between AD patients and controls in a meta-analysis [19]. Therefore, we speculated that the positive correlation of Aβ_1–43_ with Aβ_1–42_, but not with Aβ_1–40_ (except in *PSEN1* p.E318G carriers), further supports the involvement of Aβ_1–43_ in AD. It is however still unclear why Aβ_1–40_ would be unaltered and lacking of correlation with Aβ_1–43_ in the mutation carrier groups, as it is related to the process of Aβ_1–43_. It is plausible that a possible decrease of Aβ_1–40_ in CSF would only be visible or measurable during the earlier stages of the disease when mostly all neurons are still intact, rather than in later stages (when it is normally measured), when Aβ production is higher but less neurons are preserved. Alternatively, it is possible that the cleavage of the C99 fragment by γ-secretase may be heavily impaired at the third cleavage step when Aβ_1–40_ is generated from Aβ_1–43_, as suggested by Kakuda et al. [47] for the pathogenic *PSEN1* p.R278I, which would result in negligible levels of Aβ_1–40_ and unusually high levels of Aβ_1–43_. Furthermore, a recent study found a correlation between Aβ peptide length and plaque load (Aβ_1–43_ > Aβ_1–42_ > Aβ_1–40_), indicating that longer Aβ peptides have an increased tendency towards accumulation in the brain [23], thus explaining their lower CSF levels. Further analysis of the shorter Aβ species Aβ_1–41_, Aβ_1–37_ (from Aβ_1–43_) Aβ_1–38_ (from Aβ_1–42_) and Aβ_1–34_ (from either Aβ_1–43_ or Aβ_1–42_) are however needed to better clarify the role of longer Aβ peptides in AD and the pathogenic events linked to Aβ process, degradation and clearance in the presence of *APP* and *PSENs* mutations [9–11].

The production of longer Aβ peptides (> Aβ_1–42_), including Aβ_1–43_, in the pathogenesis of AD is receiving increased attention [23, 25, 26, 29, 48]. Studies showed that the *PSEN1* p.L435F produces primarily Aβ_1–43_, which was detected in the brain plaques [49] and induced pluripotent stem cell (iPSC) neurons [50] of patients carrying this mutation. Another work suggested that the *APP* mutations affect the endopeptidase activity of γ-secretase, leading to Aβ_1–42_ formation, while *PSEN1* mutations inhibit its carboxypeptidase activity, releasing multiple longer peptides including Aβ_1–42_ and Aβ_1–43_ [25]. Unfortunately, the number of mutations we investigated was not sufficient to make similar observations.

Based on the CSF biomarker analyses performed, some of the *PSENs* VUS mutations analysed showed biomarker levels comparable to the known pathogenic mutations: for instance, the *PSEN1* p.40del, which was described for the first time in an EOAD patient with prominent frontal features and no family history of dementia [51]. This variant was considered “most likely benign” or causing a small increase in risk based on its frequency in gnomAD [52]. However, an in vitro assay, testing the mutant *PSEN1* ability to cleave the APP-C99 fragment, revealed a robust decrease in Aβ_1–42_ production and undetectable levels of Aβ_1–40_ [8]. Our data is in favour of an involvement of *PSEN1* p.40del in Aβ alteration, showing decreased CSF levels of both Aβ_1–43_ and Aβ_1–42_ and unaltered Aβ_1–40_ levels in the patient carrier, and also in line with a recent study showing increased Aβ_1–42_ and unaltered Aβ_1–40_ in the medium collected from mice neuro-2A cells transfected with the plasmid containing the *PSEN1* p.40del mutation [53]. The *PSEN2* p.S130L variant was first reported in an Italian family, but segregation with disease could not be determined [54]. Another study described a carrier of this variant who had an autopsy-confirmed AD diagnosis [55]. The pathogenicity of *PSEN2* p.S130L is currently unclear, since it did not affect Aβ_1–42_ levels or Aβ_1–42_/Aβ_1–40_ ratio in in vitro experiments [56]. According to an algorithm proposed by Guerreiro et al. [57], this variant has been considered “probably pathogenic.” In line with this classification, we found a significant reduction of both Aβ_1–43_/Aβ_1–40_ and Aβ_1–42_/Aβ_1–40_ ratios in the patient carrier, pointing out to a possible pathogenicity of *PSEN2* p.S130L. Finally, the carrier of the novel *PSEN2* p.T153S variant also had reduced CSF levels of Aβ_1–43_ and Aβ_1–42_, comparable to the known pathogenic mutations. In light of these data, we could consider those mutations as “likely pathogenic”, since they seem to affect or interfere with Aβ production. Furthermore, it is important to note that the three mutation carriers were all *APOE* ε4 negative, meaning that the detected Aβ alteration is likely due to the mutations themselves without an effect of *APOE*. For the *APP* variants, instead, we could not drive conclusions on the variants pathogenicity. In fact, all the *APP* variants analysed were located outside the pathogenic exons 16–17, encoding for the Aβ domain. Furthermore, two carriers of *APP* variants were considered controls at the moment of inclusion: control 3 carried the *APP* p.S198P variant and CSF analysis did not reveal Aβ alterations; control 4, carrying the *APP* p.R328W and having altered CSF marker levels, developed vascular dementia at follow-up. Of note, control 3 but not control 4 was *APOE* ε4 negative. We would like to stress the importance to further investigate a possible interference of the *APP* variants outside the Aβ domain in AD. There was variability of Aβ_1–43_ CSF levels between the carriers of the same mutations (*PSEN1* p.A79V, *PSEN1* C263F, *APP* R328W, *APP* E599K, *PSEN2* R62H, Fig. 2; Table 2). We initially hypothesized an effect of *APOE* ε4 on the decreased Aβ_1–43_ CSF levels as already demonstrated for Aβ [58]. This seemed a valid hypothesis for all mutations but one (*PSEN1* p.C263F, Fig. 2; Table 2). However, a recent study showed a reduced influence of *APOE* on Aβ_1–43_ aggregation in cerebrovascular cells [48].

### Aβ_1–43_ CSF levels in *PSEN1* p.E318G carriers

To better understand the role of *PSEN1* p.E318G on AD pathology, we investigated the CSF Aβ_1–43_ levels in carriers, detecting a reduction. This *PSEN1* variant is suggested to be an AD risk modifier as it was associated with high levels of P-tau181P and T-tau [59, 60]. Moreover, carriers of both *APOE* ε4 and *PSEN1* p.E318G variant were reported to have a higher risk of developing LOAD than *APOE* ε4 carriers without the *PSEN1* variant [59, 60]. In our study, however, the *PSEN1* p.E318G variant did not show an association with AD regardless of *APOE* ε4 genotype, which is in line with the study of Hippen et al. [61]. A recent analysis of CSF Aβ_1–42_, T-tau and P-tau181 in asymptomatic p.E318G carriers of a large LOAD Italian family did not reveal any changes in CSF markers [62]. The levels of Aβ_1–43_ were not measured and therefore not allowing a direct comparison.

### Soluble species sAPPα and sAPPβ

We also detected reduced levels of sAPPα and sAPPβ in the three carrier groups analysed compared to controls, but statistical significance was not detected in the group of the known pathogenic mutations. Our results are different from other studies where no difference in sAPPα and sAPPβ levels was observed between patients and controls [19]. Of note, in these studies, the mutation status of the patients was not described [19].

We report that both sAPPα and sAPPβ positively correlated with Aβ_1–43_ and Aβ_1–42_ only in the control group but not in the mutation carriers. This result is in line with another study where the correlation between Aβ_1–42_ and sAPPα or sAPPβ in AD patients was not significant [63]. We cannot explain why both sAPPα and sAPPβ levels would be reduced in the mutation carriers and correlate with the control group. In fact, sAPPα and sAPPβ should not be affected by the presence of AD mutations, which affect γ-secretase activity, while sAPPα and sAPPβ are respectively generated by α- and β-secretases in the earlier steps, before Aβ production. However, the data of CSF sAPPα and sAPPβ levels in AD are unclear [19] and there are no studies so far that have measured CSF sAPPα and sAPPβ in AD mutation carriers. The possible involvement of sAPP fragments in AD is still not fully understood [18], and the interpretation of the results remains therefore challenging. It is suggested that sAPPα enhances neurogenesis and has a neuro-protective role, and it is involved in neurotransmission and synaptic plasticity [64]. A recent study, however, showed that all fragments of sAPP acted as agonists of a specific GABA receptor (GABAB-R1a) and would therefore explain their role in neurotransmission and plasticity [65]. In fact, a fragment derived from sAPPα inhibited synaptic transmission in mouse hippocampus. Additional studies in carriers of AD mutations are essential to fully understand the involvement of sAPP in AD.

### Limitations

A potential limitation of this study is the small cohort size used to analyse Aβ_1–43_ and the other markers in CSF of *APP* and *PSENs* mutation carriers, despite the genetic screening was performed in a relatively large AD population. As we know, *APP* and *PSENs* mutation carriers are extremely rare [4], as well as the availability of their biomaterials. Therefore, a replication study was not performed. The availability of a larger control cohort allowed us to reach satistical significance when analysing Aβ_1–43_ CSF levels. Unfortunately, age at onset, gender and *APOE* genotype could not be used as covariates in the analysis, because the number of mutation carriers was not sufficient for the statistical test. Our pilot study is nevertheless relevant because it highlights the involvement of Aβ_1–43_ in AD and adds missing information regarding specific mutations to very recent studies published during these last 2 years [23, 25, 48]. As CSF Aβ_1–43_ has never been studied before in CSF of *APP* and *PSENs* mutation carriers, the results need to be interpreted with caution and more studies are needed to replicate these findings in larger cohorts. Analysis of the shorter Aβ peptides (e.g. Aβ_1–34_, Aβ_1–37_, Aβ_1–38_, Aβ_1–41_) could provide a more comprehensive interpretation of our results. Lastly, in our study, it was not possible to assess the difference between *PSEN1* and *APP* mutations in affecting γ-secretase and Aβ generation [25]; therefore, this aspect warrants further follow-up, also in terms of interpretation of novel and VUS mutations in the AD genes.

## Conclusions

In this study, CSF levels of the long Aβ peptide Aβ_1–43_ were investigated for the first time in carriers of known pathogenic and unclear (VUS) *APP* and *PSENs* mutations. We observed a significant reduction of CSF Aβ_1–43_ levels and a positive correlation with Aβ_1–42_ in all mutation carrier groups. We suggested the re-classification of three VUS into “likely pathogenic”, as their biomarker levels were comparable to the known pathogenic mutations. We added important information on the debatable genetic modifier *PSEN1* p.E318G and we were able to clarify the role of *PSEN1* p.G183V, using ONT long-read sequencing, considered so far pathogenic, but probably not involved in AD.

From a clinical perspective, our data could prove useful. A recent study showed that Aβ43 was cleared more than Aβ42 in plaques of patients treated with Aβ immunotherapy [23]. These are important data that open new possibilities for personalized medicine in patients with AD, who have a high Aβ_1–43_ load in the brain. For these reasons, Aβ_1–43_ could be considered an added AD biomarker together with the others already in use. Despite the small study cohort, the data presented here corroborate previous findings on Aβ_1–43_ [21, 25, 26, 29, 49], suggesting a possible involvement of longer Aβ peptides in the AD pathophysiology.

CSF levels of sAPPα and sAPPβ were also analysed for the first time in AD mutation carriers, where their levels were reduced compared to controls. Based on the recent analysis on sAPP fragments as regulators of (GABA) neurotransmission, further investigation in AD is important to study more in detail this pathway to open new venues for therapy strategies.

Finally, functional work using patient biomaterials can prove valuable to better understand the pathogenicity of unclear AD mutations. This will be useful for patient stratification for clinical trials, genetic counselling and therapy development.

## Supplementary information

**Additional file 1: Table S1.** List of the identified *APP*, *PSEN1* and *PSEN2* mutations. **Table S2.** Diagnostic accuracy of the different markers and ratios to discriminate between controls and mutation carriers, measured by ROC curve analysis. **Table S3.** Descriptive features of *PSEN1* p.E318G mutation carriers with CSF. **Figure S1.** Neuropathology of Patient 16. Right lateral (A) and right medial (B) hemispheres showing brain atrophy. Atrophy can be observed also at the ventricles and thalamus (C). The 4G8 staining shows amyloid plaques in the hippocampus CA4 (D). Classic neurofibrillary tangles are present in the hippocampal CA4 (AT8 stain) (E). **Figure S2.** Area under the curve (AUC) calculated for the three mutation carrier groups compared to controls of Aβ_1-43_, Aβ_1-43_/Aβ_1-40_, Aβ_1-42_, Aβ_1-42_/Aβ_1-40_, Aβ_1-40_ and Aβ_1-43_/Aβ_1-42_. AUC are calculated for the know pathogenic (red), VUS (orange) and *PSEN1* p.E318G (green) mutation carrier groups compared to the control group. The AUC values and the ones for sensitivity and specificity are listed in Table S2. **Figure S3**. Area under the curve (AUC) calculated for the three mutation carrier groups compared to controls of sAPPα, sAPPβ, Aβ1_-43_/sAPPα and Aβ_1-43_/sAPPβ. AUC are calculated the know pathogenic (red), VUS (orange) and *PSEN1* p.E318G (green) mutation carrier groups compared to the control group. The AUC values and the ones for sensitivity and specificity are listed in Table S2. **Figure S4**. Transcript analysis of PSEN1 in Patient 16. The bar graph shows the relative quantifications of exon 6-7 in the double carrier (Patient 16; *PSEN1* p.G183V, *PSEN1* p.P49L), the single carrier (sibling of Patient 16; *PSEN1* p.G183V), and 4 non-carriers lymphoblast cells CHX treated (CHX) and untreated (UNT). Relative quantifications of splice junctions were calculated by dividing the number of junction-supporting reads by the total number of reads spanning the PSEN1 transcript. The quantifications for both CHX and UNT of the non-carriers are reported as averages (values of SD for CHX ± 0,001052869 and for UNT ± 0,000671837).

## Data Availability

All data relevant to the study are included in the research paper or as supplementary information. Additional information will be shared by the corresponding authors upon reasonable request.

## Abbreviations

AD: Alzheimer’s disease
AAI: Age at inclusion
AAO: Age at onset
APOE: Apolipoprotein E
APP: Amyloid precursor protein
AUC: Area under the curve
Aβ: Amyloid-β
CJD: Creutzfeldt-Jakob disease
CSF: Cerebrospinal fluid
CV: Coefficient of variation
DLB: Dementia with Lewy bodies
ELISA: Enzyme-linked immunosorbent assay
EOAD: Early-onset Alzheimer
FTD: Frontotemporal dementia
gnomAD: Genome Aggregation database
iPSC: Induced pluripotent stem cells
LOAD: Late-onset Alzheimer
MAF: Minor allele frequency
MAQ: Multiplex amplicon quantification
MCI: Mild cognitive impairment
MRI: Magnetic resonance imaging
NINCDS-ADRDA: National Institute of Neurological and Communication Disorders and Stroke Alzheimer’s disease and Related Disorders Association criteria
NFTs: Neurofibrillary tangles
NIA-AA: National Institute on Aging–Alzheimer’s Association
NMD: Non-sense-mediated mRNA decay control mechanism
P-tau181: Tau phosphorylated at threonine 181
PSEN1: Presenilin 1
PSEN2: Presenilin 2
ROC: Receiver operating characteristic
sAPPα: α cleaved soluble amyloid precursor protein
sAPPβ: β cleaved soluble amyloid precursor protein
SPECT: Single-photon emission computed tomography
STR: Short tandem repeat
T-tau: Tau protein
VaD: Vascular dementia
VUS: Variants of uncertain significance

## References

[1] Prince PM, Wimo A, Guerchet M, Ali GM, Wu YT, Prina M. The global impact of dementia. World Alzheimer Report. 2015.

[2] Cruchaga C, Del-Aguila JL, Saef B, Black K, Fernandez MV, Budde J (2018). Polygenic risk score of sporadic late-onset Alzheimer’s disease reveals a shared architecture with the familial and early-onset forms. Alzheimer’s Dement.

[3] Brouwers N, Sleegers K, Van Broeckhoven C (2008). Molecular genetics of Alzheimer’s disease: an update. Ann Med.

[4] Cacace R, Sleegers K, Broeckhoven C Van. Molecular genetics of early-onset Alzheimer disease revisited. Alzheimer’s Dement. 2016;12:733–48. 10.1016/j.jalz.2016.01.012 27016693

[5] Cruchaga C, Chakraverty S, Mayo K, Vallania FLM, Mitra RD, Faber K, et al. Rare variants in APP, PSEN1 and PSEN2 increase risk for AD in late-onset Alzheimer’s disease families. PLoS One. 2012;7:e31039. 10.1371/journal.pone.0031039 PMC3270040 22312439

[6] Veugelen S, Saito T, Saido TC, Chávez-Gutiérrez L, De Strooper B (2016). Familial Alzheimer’s disease mutations in presenilin generate amyloidogenic Aβ peptide seeds. Neuron Cell Press.

[7] Hyman BT, Phelps CH, Beach TG, Bigio EH, Cairns NJ, Carrillo MC (2012). National Institute on Aging-Alzheimer’s Association guidelines for the neuropathologic assessment of Alzheimer’s disease. Alzheimers Dement.

[8] Sun L, Zhou R, Yang G, Shi Y (2017). Analysis of 138 pathogenic mutations in presenilin-1 on the in vitro production of Aβ42 and Aβ40 peptides by γ-secretase. Proc Natl Acad Sci U S A.

[9] Zou K, Liu J, Watanabe A, Hiraga S, Liu S, Tanabe C (2013). Aβ43 is the earliest-depositing Aβ species in APP transgenic mouse brain and is converted to Aβ41 by two active domains of ACE. Am J Pathol Am J Pathol.

[10] Olsson F, Schmidt S, Althoff V, Munter LM, Jin S, Rosqvist S (2014). Characterization of intermediate steps in amyloid beta (Aβ) production under near-native conditions. J Biol Chem.

[11] Liebsch F, Kulic L, Teunissen C, Shobo A, Ulku I, Engelschalt V, et al. Aβ34 is a BACE1-derived degradation intermediate associated with amyloid clearance and Alzheimer’s disease progression. Nat Commun. 2019;10:2240. 10.1038/s41467-019-10152-w PMC6527709 31110178

[12] Struyfs H, Van Broeck B, Timmers M, Fransen E, Sleegers K, Van Broeckhoven C (2015). Diagnostic accuracy of cerebrospinal fluid amyloid-β isoforms for early and differential dementia diagnosis. J Alzheimer’s Dis.

[13] Sunderland T, Linker G, Mirza N, Putnam KT, Friedman DL, Kimmel LH (2003). Decreased β-amyloid _1-42_ and increased tau levels in cerebrospinal fluid of patients with Alzheimer disease. JAMA.

[14] Fagan AM, Mintun MA, Mach RH, Lee S-Y, Dence CS, Shah AR (2006). Inverse relation between in vivo amyloid imaging load and cerebrospinal fluid Abeta42 in humans. Ann Neurol.

[15] Buchhave P, Minthon L, Zetterberg H, Wallin ÅK, Blennow K, Hansson O (2012). Cerebrospinal fluid levels ofβ-amyloid 1–42, but not of tau, are fully changed already 5 to 10 years before the onset of Alzheimer dementia. Arch Gen Psychiatry.

[16] Lauridsen C, Sando SB, Shabnam A, Møller I, Berge G, Grøntvedt GR (2016). Cerebrospinal fluid levels of amyloid beta 1–43 in patients with amnestic mild cognitive impairment or early Alzheimer’s disease: a 2-year follow-up study. Front Aging Neurosci.

[17] Bjerke M, Engelborghs S. Cerebrospinal fluid biomarkers for early and differential Alzheimer’s disease diagnosis. Perry G, Avila J, Tabaton M, Zhu X, editors. J Alzheimer’s Dis; 2018;62:1199–1209. 10.3233/JAD-170680 PMC5870045 29562530

[18] Morris GP, Clark IA, Vissel B, Hardy J, Mayer J, Prusiner S (2014). Inconsistencies and controversies surrounding the amyloid hypothesis of Alzheimer’s disease. Acta Neuropathol Commun.

[19] Olsson B, Lautner R, Andreasson U, Öhrfelt A, Portelius E, Bjerke M, et al. CSF and blood biomarkers for the diagnosis of Alzheimer’s disease: a systematic review and meta-analysis. Lancet Neurol. 2016;15:673–84. 10.1016/S1474-4422(16)00070-3 27068280

[20] Scheuner D, Eckman C, Jensen M, Song X, Citron M, Suzuki N (1996). Secreted amyloid β–protein similar to that in the senile plaques of Alzheimer’s disease is increased in vivo by the presenilin 1 and 2 and APP mutations linked to familial Alzheimer’s disease. Nat Med.

[21] Saito T, Suemoto T, Brouwers N, Sleegers K, Funamoto S, Mihira N (2011). Potent amyloidogenicity and pathogenicity of Aβ43. Nat Neurosci.

[22] Welander H, Frånberg J, Graff C, Sundström E, Winblad B, Tjernberg LO (2009). Aβ43 is more frequent than Aβ40 in amyloid plaque cores from Alzheimer disease brains. J Neurochem.

[23] Jäkel L, Boche D, Nicoll JAR, Verbeek MM (2019). Aβ43 in human Alzheimer’s disease: effects of active Aβ42 immunization. Acta Neuropathol Commun.

[24] Sandebring A, Welander H, Winblad B, Graff C, Tjernberg LO (2013). The pathogenic Ab43 is enriched in familial and sporadic Alzheimer disease. PLoS One.

[25] Arber C, Toombs J, Lovejoy C, Ryan NS, Paterson RW, Willumsen N, et al. Familial Alzheimer’s disease patient-derived neurons reveal distinct mutation-specific effects on amyloid beta. Mol Psychiatry. 2019. 10.1038/s41380-019-0410-8. 10.1038/s41380-019-0410-8 PMC7577860 30980041

[26] Szaruga M, Munteanu B, Lismont S, Veugelen S, Horré K, Mercken M (2017). Alzheimer’s-causing mutations shift Aβ length by destabilizing γ-secretase-Aβn interactions. Cell.

[27] Cruts M, Theuns J, Van Broeckhoven C (2012). Locus-specific mutation databases for neurodegenerative brain diseases. Hum Mutat.

[28] Bruggink KA, Kuiperij BH, Claassen JAHR, Verbeek MM. The diagnostic value of CSF amyloidβ43 in differentiation of dementia syndromes. Curr Alzheimer Res. 2013;10:1034–40. 10.2174/15672050113106660168 24156268

[29] Lauridsen C, Sando SB, Møller I, Berge G, Pomary PK, Grøntvedt GR (2017). Cerebrospinal fluid Aβ43 is reduced in early-onset compared to late-onset Alzheimer’s disease, but has similar diagnostic accuracy to Aβ42. Front Aging Neurosci.

[30] McKhann GM, Knopman DS, Chertkow H, Hyman BT, Jack CR, Kawas CH (2011). The diagnosis of dementia due to Alzheimer’s disease: recommendations from the National Institute on Aging-Alzheimer’s Association workgroups on diagnostic guidelines for Alzheimer’s disease. Alzheimers Dement.

[31] Folstein MF, Folstein SE, McHugh PR. “Mini-mental state”. A practical method for grading the cognitive state of patients for the clinician. J Psychiatr Res 1975;12:189–198. 10.1016/0022-3956(75)90026-6 1202204

[32] Nasreddine ZS, Phillips NA, Bédirian V, Charbonneau S, Whitehead V, Collin I (2005). The Montreal Cognitive Assessment, MoCA: a brief screening tool for mild cognitive impairment. J Am Geriatr Soc.

[33] Timmers M, Barão S, Van Broeck B, Tesseur I, Slemmon J, De Waepenaert K (2017). BACE1 dynamics upon inhibition with a BACE inhibitor and correlation to downstream Alzheimer’s disease markers in elderly healthy participants. J Alzheimer’s Dis.

[34] Perrone F, Cacace R, Van Mossevelde S, Van den Bossche T, De Deyn PP, Cras P, et al. Genetic screening in early-onset dementia patients with unclear phenotype: relevance for clinical diagnosis. Neurobiol Aging. 2018;69:292e7–14. 10.1016/j.neurobiolaging.2018.04.015 29859640

[35] Karczewski KJ, Francioli LC, Tiao G, Cummings BB, Alföldi J, Wang Q, et al. The mutational constraint spectrum quantified from variation in 141,456 humans. Nature. 2020;581:434–43. 10.1038/s41586-020-2308-7 PMC7334197 32461654

[36] Weckx S, Del-Favero J, Rademakers R, Claes L, Cruts M, De Jonghe P (2005). novoSNP, a novel computational tool for sequence variation discovery. Genome Res.

[37] Sleegers K, Brouwers N, Gijselinck I, Theuns J, Goossens D, Wauters J (2006). APP duplication is sufficient to cause early onset Alzheimer’s dementia with cerebral amyloid angiopathy. Brain.

[38] Le Bastard N, Aerts L, Sleegers K, Martin J-J, Van Broeckhoven C, De Deyn PP (2013). Longitudinal stability of cerebrospinal fluid biomarker levels: fulfilled requirement for pharmacodynamic markers in Alzheimer’s disease. J Alzheimer’s Dis.

[39] De Roeck A, Van den Bossche T, van der Zee J, Verheijen J, De Coster W, Van Dongen J, et al. Deleterious ABCA7 mutations and transcript rescue mechanisms in early onset Alzheimer’s disease. Acta Neuropathol. 2017;134:475–87. 10.1007/s00401-017-1714-x PMC5563332 28447221

[40] Untergasser A, Nijveen H, Rao X, Bisseling T, Geurts R, Leunissen JAM (2007). Primer3Plus, an enhanced web interface to Primer3. Nucleic Acids Res.

[41] Li H. Minimap2: pairwise alignment for nucleotide sequences. Birol I, editor. Bioinformatics. 2018;34:3094–3100. 10.1093/bioinformatics/bty191 PMC6137996 29750242

[42] Dermaut B, Kumar-singh S, Engelborghs S, Theuns J, Rademakers R, Saerens J (2004). A novel presenilin 1 mutation associated with Pick ’s disease but not β-amyloid plaques.

[43] Watanabe H, Xia D, Kanekiyo T, Kelleher RJ, Shen J (2012). Familial frontotemporal dementia-associated presenilin-1 c.548G>T mutation causes decreased mRNA expression and reduced presenilin function in knock-in mice. J Neurosci.

[44] Conidi ME, Bernardi L, Puccio G, Smirne N, Muraca MG, Curcio SAM (2015). Homozygous carriers of APP A713T mutation in an autosomal dominant Alzheimer disease family. Neurology.

[45] Zee J Van Der, Van C. Invited Article : The Alzheimer disease – frontotemporal lobar degeneration spectrum. 2008;1191–7. 10.1212/01.wnl.0000327523.52537.86 18838666

[46] Chávez-Gutiérrez L, Bammens L, Benilova I, Vandersteen A, Benurwar M, Borgers M (2012). The mechanism of γ-secretase dysfunction in familial Alzheimer disease. EMBO J.

[47] Kakuda N, Shoji M, Arai H, Furukawa K, Ikeuchi T, Akazawa K (2012). Altered γ-secretase activity in mild cognitive impairment and Alzheimer’s disease. EMBO Mol Med.

[48] Jäkel L, Biemans EALM, Klijn CJM, Kuiperij HB, Verbeek MM. Reduced influence of apoE on Aβ43 aggregation and reduced vascular Aβ43 toxicity as compared with Aβ40 and Aβ42. Mol Neurobiol. 2020;57:2131–41. 10.1007/s12035-020-01873-x PMC7118029 31953617

[49] Kretner B, Trambauer J, Fukumori A, Mielke J, Kuhn P-H, Kremmer E (2016). Generation and deposition of Aβ43 by the virtually inactive presenilin-1 L435F mutant contradicts the presenilin loss-of-function hypothesis of Alzheimer’s disease. EMBO Mol Med.

[50] Oakley DH, Chung M, Klickstein N, Commins C, Hyman BT, Frosch MP (2020). The Alzheimer disease-causing presenilin-1 L435F mutation causes increased production of soluble Aβ43 species in patient-derived iPSC-neurons, closely mimicking matched patient brain tissue. J Neuropathol Exp Neurol.

[51] Nygaard HB, Lippa CF, Mehdi D, Baehring JM (2014). A novel presenilin 1 mutation in early-onset Alzheimer’s disease with prominent frontal features. Am J Alzheimers Dis Other Dement.

[52] Koriath C, Kenny J, Adamson G, Druyeh R, Taylor W, Beck J, et al. Predictors for a dementia gene mutation based on gene-panel next-generation sequencing of a large dementia referral series. Mol Psychiatry. 2018. 10.1038/s41380-018-0224-0. 10.1038/s41380-018-0224-0 PMC6330090 30279455

[53] Hsu S, Pimenova AA, Hayes K, Villa JA, Rosene MJ, Jere M (2020). Systematic validation of variants of unknown significance in APP, PSEN1 and PSEN2. Neurobiol Dis.

[54] Tedde A, Nacmias B, Ciantelli M, Forleo P, Cellini E, Bagnoli S (2003). Identification of new presenilin gene mutations in early-onset familial Alzheimer disease. Arch Neurol.

[55] Sassi C, Guerreiro R, Gibbs R, Ding J, Lupton MK, Troakes C (2014). Investigating the role of rare coding variability in Mendelian dementia genes (APP, PSEN1, PSEN2, GRN, MAPT, and PRNP) in late-onset Alzheimer’s disease. Neurobiol Aging.

[56] Walker ES, Martinez M, Brunkan AL, Goate A (2005). Presenilin 2 familial Alzheimer’s disease mutations result in partial loss of function and dramatic changes in Abeta 42/40 ratios. J Neurochem.

[57] Guerreiro RJ, Baquero M, Blesa R, Boada M, Brás JM, Bullido MJ (2010). Genetic screening of Alzheimer’s disease genes in Iberian and African samples yields novel mutations in presenilins and APP. Neurobiol Aging.

[58] Huang YWA, Zhou B, Wernig M, Südhof TC (2017). ApoE2, ApoE3, and ApoE4 differentially stimulate APP transcription and Aβ secretion. Cell.

[59] Benitez BA, Karch CM, Cai Y, Jin SC, Cooper B, Carrell D, et al. The PSEN1, p.E318G variant increases the risk of Alzheimer’s disease in APOE-ε4 carriers. Myers AJ, editor. PLoS Genet. 2013;9:e1003685. 10.1371/journal.pgen.1003685 PMC3750021 23990795

[60] Nho K, Horgusluoglu E, Kim S, Risacher SL, Kim D, Foroud T (2016). Integration of bioinformatics and imaging informatics for identifying rare PSEN1 variants in Alzheimer’s disease. BMC Med Genomics.

[61] Hippen AA, Ebbert MTW, Norton MC, Tschanz JAT, Munger RG, Corcoran CD (2016). Presenilin E318G variant and Alzheimer’s disease risk: the Cache County study. BMC Genomics.

[62] Artuso V, Benussi L, Ghidoni R, Moradi-Bachiller S, Fusco F, Curtolo S, et al. Asymptomatic carriers of presenilin-1 E318G variant show no cerebrospinal fluid biochemical signs suggestive of Alzheimer’s disease in a family with late-onset dementia. Curr Alzheimer Res. 2018;16:1–7. 10.2174/1567205015666181031150345 30381075

[63] Araki W, Hattori K, Kanemaru K, Yokoi Y, Omachi Y, Takano H (2017). Re-evaluation of soluble APP-α and APP-β in cerebrospinal fluid as potential biomarkers for early diagnosis of dementia disorders. Biomark Res.

[64] Hick M, Herrmann U, Weyer SW, Mallm JP, Tschäpe JA, Borgers M (2015). Acute function of secreted amyloid precursor protein fragment APPsα in synaptic plasticity. Acta Neuropathol.

[65] Rice HC, De Malmazet D, Schreurs A, Frere S, Van Molle I, Volkov AN, et al. Secreted amyloid-b precursor protein functions as a GABA B R1a ligand to modulate synaptic transmission. Science. 2019:363;4827. 10.1126/science.aao4827 PMC6366617 30630900

