# Supplementary material for: Amyloid-β1–43 cerebrospinal fluid levels and the interpretation of APP, PSEN1 and PSEN2 mutations
Source: Alzheimers Res Ther. 2020 Sep 11;12:108. doi: 10.1186/s13195-020-00676-5 (PMC7488767; doi:10.1186/s13195-020-00676-5)
Supplement: Supplementary file 1 — Additional file 1: Table S1. List of the identified APP, PSEN1 and PSEN2 mutations. Table S2. Diagnostic accuracy of the different markers and ratios to discriminate between controls and mutation carriers, measured by ROC curve analysis. Table S3. Descriptive features of PSEN1 p.E318G mutation carriers with CSF. Figure S1. Neuropathology of Patient 16. Right lateral (A) and right medial (B) hemispheres showing brain atrophy. Atrophy can be observed also at the ventricles and thalamus (C). The 4G8 staining shows amyloid plaques in the hippocampus CA4 (D). Classic neurofibrillary tangles are present in the hippocampal CA4 (AT8 stain) (E). Figure S2. Area under the curve (AUC) calculated for the three mutation carrier groups compared to controls of Aβ1-43, Aβ1-43/Aβ1-40, Aβ1-42, Aβ1-42/Aβ1-40, Aβ1-40 and Aβ1-43/Aβ1-42. AUC are calculated for the know pathogenic (red), VUS (orange) and PSEN1 p.E318G (green) mutation carrier groups compared to the control group. The AUC values and the ones for sensitivity and specificity are listed in Table S2. Figure S3. Area under the curve (AUC) calculated for the three mutation carrier groups compared to controls of sAPPα, sAPPβ, Aβ1-43/sAPPα and Aβ1-43/sAPPβ. AUC are calculated the know pathogenic (red), VUS (orange) and PSEN1 p.E318G (green) mutation carrier groups compared to the control group. The AUC values and the ones for sensitivity and specificity are listed in Table S2. Figure S4. Transcript analysis of PSEN1 in Patient 16. The bar graph shows the relative quantifications of exon 6-7 in the double carrier (Patient 16; PSEN1 p.G183V, PSEN1 p.P49L), the single carrier (sibling of Patient 16; PSEN1 p.G183V), and 4 non-carriers lymphoblast cells CHX treated (CHX) and untreated (UNT). Relative quantifications of splice junctions were calculated by dividing the number of junction-supporting reads by the total number of reads spanning the PSEN1 transcript. The quantifications for both CHX and UNT of the non-carriers are reported a [file 13195_2020_676_MOESM1_ESM.docx]

**Amyloid-β_1-43_ cerebrospinal fluid levels and the interpretation of *APP*, *PSEN1* and *PSEN2* mutations**

Federica Perrone^1,2,3^, Maria Bjerke^2,4,5^, Elisabeth Hens^1,2,3,6,7,8^, Anne Sieben^1,2,9^, Maarten Timmers^4,10^, Arne De Roeck^1,2,3^, Rik Vandenberghe^11,12^, Kristel Sleegers^1,2,3^, Jean-Jacques Martin^2^, Peter P. De Deyn^2,3,6^, Sebastiaan Engelborghs^4,8^, Julie van der Zee^1,2,3^, Christine Van Broeckhoven^1,2,3*^, Rita Cacace^1,2,3*^ on behalf of the BELNEU Consortium

**Affiliation of all authors:**

^1^Neurodegenerative Brain Diseases Group, VIB Center for Molecular Neurology, Antwerp, Belgium

^2^Institute Born-Bunge, Antwerp, Belgium

^3^Department of Biomedical Sciences, University of Antwerp, Antwerp, Belgium

^4^Reference Centre for Biological Markers of Dementia (BIODEM), Institute Born-Bunge, University of Antwerp, Antwerp, Belgium

^5^Laboratory of Neurochemistry and Center for Neurosciences, UZ Brussel and Vrije University Brussels, Brussels, Belgium

^6^Department of Neurology and Memory Clinic, Hospital Network Antwerp, Middelheim and Hoge Beuken, Antwerp, Belgium

^7^Department of Neurology, University Hospital Antwerp, Edegem, Belgium

^8^Department of Neurology and Center for Neurosciences, UZ Brussel and Vrije University Brussels, Brussels, Belgium.

^9^Department of Neurology, University Hospital Ghent and University of Ghent, Ghent, Belgium

^10^Janssen Research and Development, Division of Janssen Pharmaceutica NV, Beerse, Belgium.

^11^Department of Neurosciences, Faculty of Medicine, KU Leuven, Louvain, Belgium

^12^Laboratory of Cognitive Neurology, Department of Neurology, University Hospitals Leuven, Louvain, Belgium

***Shared corresponding authors:**

Christine Van Broeckhoven, PhD, DSc, [christine.vanbroeckhoven@uantwerpen.vib.be](mailto:christine.vanbroeckhoven@uantwerpen.vib.be)

Rita Cacace, PhD, [rita.cacace@uantwerpen.vib.be](mailto:rita.cacace@uantwerpen.vib.be)

**Address:**

VIB Center for Molecular Neurology

University of Antwerp - CDE

Universiteitsplein 1, Parking P4, Building VA, Room VA.121, B-2610 Antwerp, Belgium

**SUPPLEMENTARY DATA**

**Table S1. List of the identified *APP*, *PSEN1* and *PSEN2* mutations**

| Gene |  | Protein change | cDNA position | Individual identifier | *APOE* | Phenotype | dbSNP id | gnomAD  (%) NFE |
| --- | --- | --- | --- | --- | --- | --- | --- | --- |
| *APP* | P | p.V717I | c.2149G>A | **Patient 1** | 33 | AD | rs63750264 | - |
|  |  | p.V717G | c.2150T>G | **Patient 2** | 34 | AD | rs63749964 | - |
|  |  | p.A713T | c.2137G>A | Control 1 | 33 | C | rs63750066 | - |
|  |  | p.E682K | c.2044G>A | **Patient 3** | 33 | AD | - | - |
|  | V | p.R100W | c.298C>T | Control 2 | 24 | C | rs200347552 | 0.003 |
|  |  | p.S198P | c.592T>C | Control 3 | 33 | C | rs145081708 | 0.01 |
|  |  | p.A201V | c.602C>T | Patient 4 | 33 | AD | rs149995579 | 0.021 |
| ` |  | p.R328W | c.982C>T | Patient 5 | 33 | AD | rs200978018 | 0.033 |
|  |  | p.R328W | c.982C>T | Control 4 | 34 | C | rs200978018 | 0.033 |
|  |  | p.R328W | c.982C>T | Control 5 | 33 | C | rs200978018 | 0.033 |
|  |  | p.R328W | c.982C>T | Control 6 | 34 | C | rs200978018 | 0.033 |
|  |  | p.R499H | c.1496G>A | Patient 6 | 34 | AD | rs751737465 | 0.001 |
|  |  | p.E599K | c.1795G>A | Patient 7 | 34 | AD | rs140304729 | 0.144 |
|  |  | p.E599K | c.1795G>A | Patient 8 | 33 | AD | rs140304729 | 0.144 |
|  |  | p.E599K | c.1795G>A | Control 7 | 33 | C | rs140304729 | 0.144 |
|  |  | p.T761A | c.2281A>G | Control 8 | 33 | C | rs766455623 | - |
|  | N | p.L5F | c.15G>C | **Patient 9** | 33 | AD | - | - |
|  |  | p.P91L | c.272C>T | Patient 10 | 34 | AD | - | - |
|  |  | p.D214G | c.641A>G | Control 9 | 33 | C | - | - |
|  |  | p.T266A | c.796A>G | Control 10 | 33 | C | - | - |
|  |  | p.G625_S628del | c.1874_1885del | Patient 11**^&^** | 33 | AD | - | - |
| *PSEN1* | P | p.A79V | c.236C>T | **Patient 12** | 34 | MXD | rs63749824 | 0.002 |
|  |  | p.A79V | c.236C>T | **Patient 13** | 33 | AD | rs63749824 | 0.002 |
|  |  | p.I143T | c.428T>C | **Patient 14** | 34 | AD | rs63750004 | - |
|  |  | p.S170F | c.509C>T | **Patient 15** | 33 | AD | rs63750577 | - |
|  |  | p.G183V | c.548G>T | Patient 16**^$^** | 34 | AD | rs63751068 | - |
|  |  | p.C263F | c.788G>T | Patient 17 | 33 | AD | rs63751102 | - |
|  |  | p.C263F | c.788G>T | **Patient 18** | 33 | AD | rs63751102 | - |
|  |  | p.C263F | c.788G>T | Patient 19 | 33 | AD | rs63751102 | - |
|  |  | p.C263F | c.788G>T | **Patient 20** | 34 | AD | rs63751102 | - |
|  |  | p.C263F | c.788G>T | **Patient 21** | 33 | AD | rs63751102 | - |
|  |  | p.P264L | c.791C>T | **Patient 22** | 33 | AD | rs63750301 | - |
|  |  | p.G266S | c.796G>A | **Patient 23** | 34 | AD | rs121917807 | - |
|  |  | p.R269H | c.806G>A | **Patient 24** | 33 | AD | rs63750900 | - |
|  |  | p.L282V | c.844C>G | **Patient 25** | 34 | AD | rs63749937 | - |
|  |  | p.G384A | c.1151G>C | **Patient 26** | 33 | AD | rs63750646 | - |
|  |  | p.G384A | c.1151G>C | **Patient 27** | 34 | AD | rs63750646 | - |
|  | V | p.D40del | c.118_120del | Patient 28 | 33 | AD | rs759538127 | 0.021 |
|  |  | p.D40del | c.118_120del | Patient 29 | 33 | AD | rs759538127 | 0.021 |
|  |  | p.P355S | c.1063C>T | Patient 11**^&^** | 33 | AD | rs376433615 | - |
|  | N | p.P49L | c.146C>T | Patient 16**^$^** | 34 | AD | - | - |
|  |  | p.G371C | c.1111G>T | Patient 30 | 34 | AD | - | - |
| *PSEN2* | V | p.G34S | c.100G>A | Control 11 | 33 | C | - | 0.001 |
|  |  | p.R62C | c.184C>T | Patient 31 | 34 | AD | rs150400387 | 0.033 |
|  |  | p.R62H | c.185G>A | Patient 32 | 33 | AD | rs58973334 | 0.232 |
|  |  | p.R62H | c.185G>A | Patient 33 | 33 | AD | rs58973334 | 0.232 |
|  |  | p.R62H | c.185G>A | Patient 34 | 34 | AD | rs58973334 | 0.232 |
|  |  | p.R71W | c.211C>T | Control 12 | 34 | C | rs140501902 | 0.392 |
|  |  | p.R71W | c.211C>T | Patient 35 | 44 | AD | rs140501902 | 0.392 |
|  |  | p.S130L | c.389C>T | Patient 36 | 33 | AD | rs63750197 | 0.117 |
|  |  | p.S130L | c.389C>T | Patient 37 | 34 | AD | rs63750197 | 0.117 |
|  |  | p.T421M | c.1262C>T | Patient 38 | 33 | AD | rs756609078 | 0.001 |
|  | N | p.I149T | c.446T>C | **Patient 39** | 33 | AD | - | - |
|  |  | p.T153S | c.458C>G | **Patient 40** | 33 | AD | - | - |
|  |  | p.G359Lfs*74 | c.1073-2delA | Patient 41 | 33 | AD | - | - |

*The carriers of APP, PSEN1 and PSEN2 variants are listed. Notes: APP p.A201V, p.E599K, PSEN2 p.R71W are labelled as benign in Alzforum (*[*www.alzforum.org/mutations*](http://www.alzforum.org/mutations)*). Patients in bold are EOAD patients (AAO range 23-65 years). Known unclear and novel variants are considered as variants of uncertain significance. The ^$^symbol indicates the double carrier of PSEN1 p.G183V and PSEN1 p.P49; the ^&^ symbol indicates the double carrier of APP p.G625_S628del and PSEN1 p.P355S. Abbreviations:; MXD, mixed (AD+Vascular); NFE, non-Finnish European; N, novel; P, pathogenic; V, variants of uncertain significance.*

**Table S2. Diagnostic accuracy of the different markers and ratios to discriminate between controls and mutation carriers, measured by ROC curve analysis**

|  | ***Pathogenic vs controls*** | | | | | ***VUS vs controls*** | | | | | ***PSEN1 p.E318G vs Controls*** | | | | |
| --- | --- | --- | --- | --- | --- | --- | --- | --- | --- | --- | --- | --- | --- | --- | --- |
|  | AUC | cut-off | sens | spec | AUC | | cut-off | sens | spec | AUC | | cut-off | sens | spec |  |
| Aβ_1-43_ | 0.923 | <22.00 | 80 | 95.31 | 0.893 | | <18.00 | 69.23 | 100 | 0.919 | | <23.00 | 90 | 95.31 |  |
| Aβ_1-42_ | 0.935 | <729.5 | 80 | 100 | 0.845 | | <865.5 | 76.92 | 98.11 | 0.902 | | <906.0 | 90.91 | 85.94 |  |
| Aβ_1-40_ | 0.646 | <4908 | 60 | 90.63 | 0.619 | | <5186 | 46.15 | 85.94 | 0.684 | | <6244 | 65 | 68.75 |  |
| sAPPα | 0.625 | <130.5 | 80.37 | 60.94 | 0.793 | | <122.5 | 84.62 | 71.88 | 0.759 | | <143.5 | 94.74 | 53.13 |  |
| sAPPβ | 0.625 | <143.5 | 80 | 57.81 | 0.789 | | <128 | 84.62 | 70.31 | 0.727 | | <154 | 100 | 54.69 |  |
| Aβ_1-43_/Aβ_1-40_ | 0.979 | <0.004 | 100 | 92.19 | 0.933 | | <0.005 | 92.31 | 78.13 | 0.919 | | <0.004 | 85 | 98.44 |  |
| Aβ_1-42_/Aβ_1-40_ | 0.959 | <0.133 | 100 | 90.63 | 0.862 | | <0.161 | 84.62 | 81.25 | 0.899 | | <0.110 | 80 | 98.44 |  |
| Aβ_1-43_/Aβ_1-42_ | 0.796 | <0.031 | 80 | 70.31 | 0.884 | | <0.020 | 76.92 | 93.75 | 0.923 | | < 0.028 | 85.63 | 90.63 |  |
| Aβ_1-43_/sAPPα | 0.868 | <0.207 | 80.37 | 79.69 | 0.725 | | <0.271 | 84.62 | 57.81 | 0.868 | | <0.180 | 85.63 | 87.5 |  |
| Aβ_1-43_/sAPPβ | 0.871 | <0.180 | 80.27 | 81.25 | 0731 | | <0.233 | 76.42 | 68.75 | 0.886 | | <0.154 | 85.63 | 95.31 |  |

*Receiver operating characteristic (ROC) curve analysis was used to assess the diagnostic utility of the three mutation carrier groups compared to controls. The optimal cut-off value (corresponding to the highest Youden´s index), the values of sensitivity and specificity are given. Abbreviations: AUC, area under the curve; sens, sensitivity; spec, specificity. Values for sensitivity and specificity are given in %. VUS: variants of uncertain significance.*

**Table S3. Descriptive features of *PSEN1 p.E318G* mutation carriers with CSF**

| **Individual identifier** | ***APOE*** | **Aβ_1-43_** | **Aβ_1-42_** | **Aβ_1-40_** | **sAPPα** | **sAPPβ** | **T-tau** | **P-tau181** |
| --- | --- | --- | --- | --- | --- | --- | --- | --- |
| **Patient 42** | 33 | 12 | 670 | 6185 | 143 | 151 | 622 | 116 |
| **Patient 43** | 34 | 19 | 903 | 5816 | 89 | 107 | NA | NA |
| **Patient 44** | 33 | 8 | 325 | 5286 | 152 | 152 | 982 | 34 |
| **Patient 45** | 23 | 19 | 700 | 7362 | 129 | 136 | 341 | 63 |
| Patient 46 | 33 | 55 | 1689 | 7514 | 33 | 37 | >1200 | 53 |
| Patient 47 | 33 | 53 | 1534 | 8004 | 97 | 112 | NA | NA |
| Patient 48 | 44 | 9 | 386 | 3905 | 105 | 90 | 533 | 46 |
| Patient 49 | 34 | 6 | 262 | 2697 | 36 | 54 | NA | NA |
| Patient 50 | 33 | 10 | 448 | 4766 | 82 | 101 | 509 | 63 |
| Patient 51 | 34 | 9 | 355 | 4156 | 75 | 107 | 862 | 77 |
| Patient 52 | 34 | 21 | 687 | 6841 | 117 | 136 | 233 | 43 |
| Patient 53 | 33 | 22 | 857 | 8869 | 136 | 145 | NA | NA |
| Patient 54 | 34 | 7 | 384 | 4781 | 143 | 143 | 382 | 62 |
| Patient 55 | 34 | 9 | 566 | 5293 | 67 | 78 | 379 | 69 |
| Patient 56 | 34 | 5 | 373 | 5080 | 107 | 126 | 370 | 61 |
| Patient 57 | 44 | 8 | 487 | 6737 | 140 | 145 | 315 | 62 |
| Patient 58 | 34 | 22 | 810 | 5648 | 125 | 145 | NA | NA |
| Patient 59 | 44 | 5 | 382 | 6218 | 119 | 151 | 535 | 68 |
| Patient 60 | 34 | 13 | 777 | 9255 | 110 | 127 | 406 | 9 |
| Patient 61 | 33 | 6 | 445 | 4987 | 88 | 101 | 485 | 91 |

*List of carriers of PSEN1 p.E183G with CSF available. Notes: all carriers had AD diagnosis. Patients in bold are EOAD patients (AAO range 62-65 years). Normal cut offs: T-tau: <297 pg/mL; P-tau181 <57 pg/mL.*

***
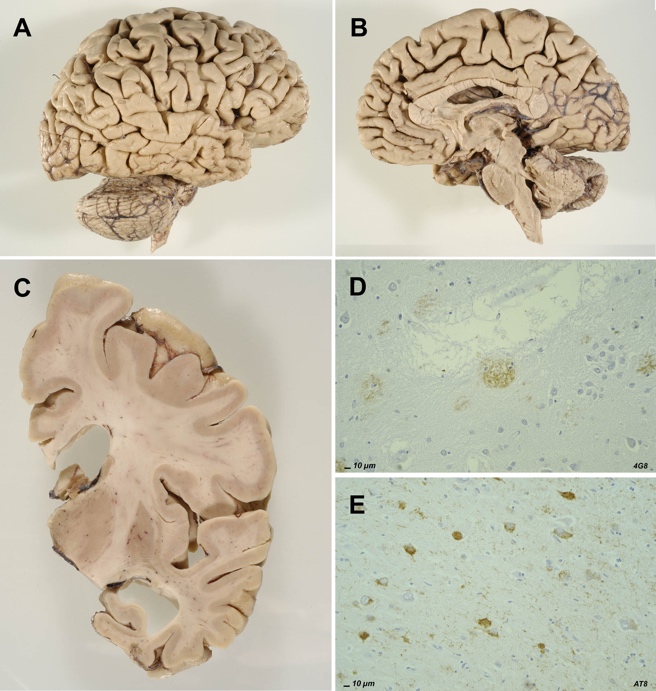
***

***Figure S1. Neuropathology of Patient 16.*** *Right lateral (A) and right medial (B) hemispheres showing brain atrophy. Atrophy can be observed also at the ventricles and thalamus (C).* *The 4G8 staining shows amyloid plaques in the hippocampus CA4 (D). Classic neurofibrillary tangles are present in the hippocampal CA4 (AT8 stain) (E).*

*
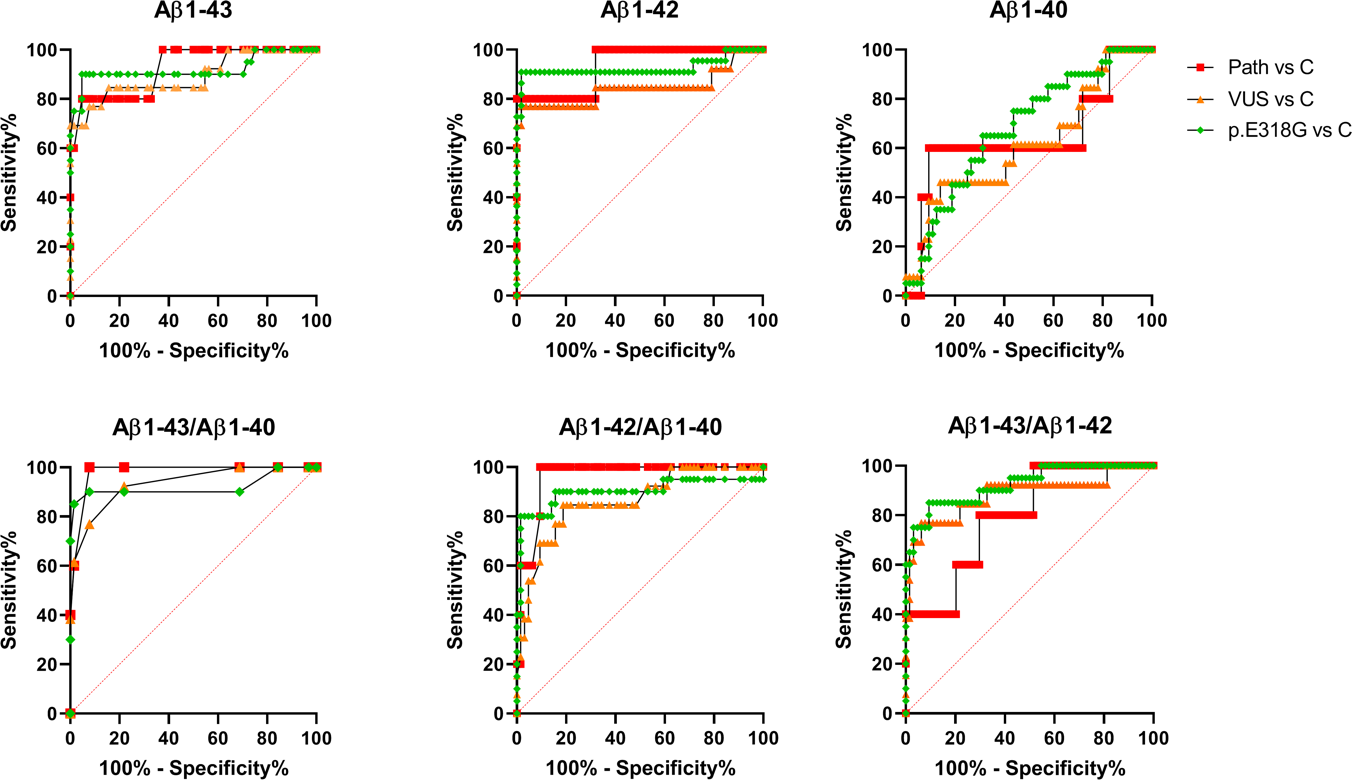
*

***Figure S2. Area under the curve (AUC) calculated for the three mutation carrier groups compared to controls of Aβ_1-43_, Aβ_1-43_/Aβ_1-40_, Aβ_1-42_, Aβ_1-42_/Aβ_1-40_, Aβ_1-40_ and Aβ_1-43_/Aβ_1-42_.*** *AUC are calculated for the know pathogenic (red), VUS (orange) and PSEN1 p.E318G (green) mutation carrier groups compared to the control group. The AUC values and the ones for sensitivity and specificity are listed in Table S2.*

***
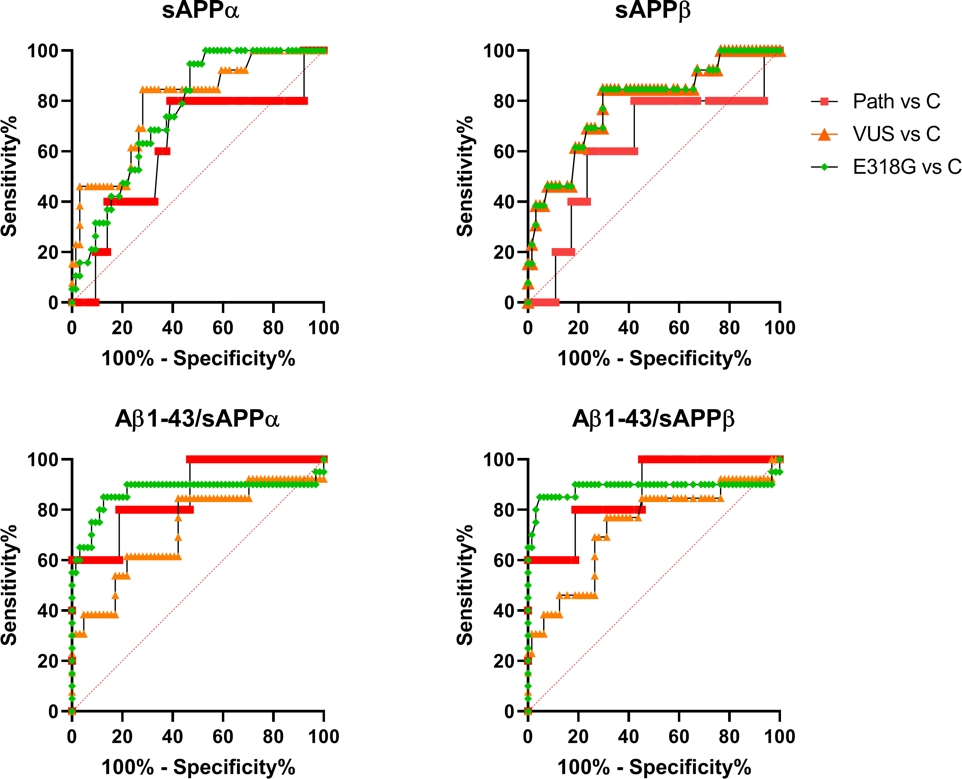
***

***Figure S3. Area under the curve (AUC) calculated for the three mutation carrier groups compared to controls of sAPPα, sAPPβ, Aβ1_-43_/sAPPα and Aβ_1-43_/sAPPβ.*** *AUC are calculated the know pathogenic (red), VUS (orange) and PSEN1 p.E318G (green) mutation carrier groups compared to the control group. The AUC values and the ones for sensitivity and specificity are listed in Table S2.*

***
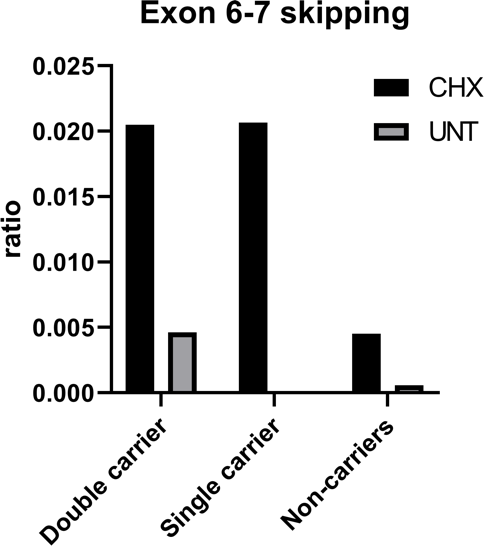
***

***Figure S4. Transcript analysis of PSEN1 in Patient 16.*** *The bar graph shows the relative quantifications of exon 6-7 in the double carrier (Patient 16; PSEN1 p.G183V, PSEN1 p.P49L), the single carrier (sibling of Patient 16; PSEN1 p.G183V), and 4 non-carriers lymphoblast cells CHX treated (CHX) and untreated (UNT). Relative quantifications of splice junctions were calculated by dividing the number of junction-supporting reads by the total number of reads spanning the PSEN1 transcript. The quantifications for both CHX and UNT of the non-carriers are reported as averages (values of SD for CHX* ± *0,001052869 and for UNT* ± *0,000671837).*
